# Polydopamine-Cloaked Nanoarchitectonics of Prussian Blue Nanoparticles Promote Functional Recovery in Neonatal and Adult Ischemic Stroke Models

**DOI:** 10.34133/bmr.0079

**Published:** 2024-09-18

**Authors:** Yijing Zhao, Cong Song, Haijun Wang, Chengcheng Gai, Tingting Li, Yahong Cheng, Junjie Liu, Yan Song, Qian Luo, Bing Gu, Weiyang Liu, Liwei Chai, Dexiang Liu, Zhen Wang

**Affiliations:** ^1^ Department of Physiology, School of Basic Medical Sciences, Cheeloo College of Medicine, Shandong University, Jinan, Shandong 250012, P.R. China.; ^2^ Medical Science and Technology Innovation Center, Shandong First Medical University, Jinan, Shandong 250117, P.R. China.; ^3^ Department of Medical Psychology and Ethics, School of Basic Medicine Sciences, Cheeloo College of Medicine, Shandong University, Jinan, Shandong 250012, P.R. China.; ^4^ Jinan Xicheng Experimental High School, Dezhou Road1999, Jinan, Shandong, P.R. China.

## Abstract

Ischemic stroke is a devastating disease and one of the leading causes of mortality worldwide. Overproduction of reactive oxygen species and inflammatory response contribute to secondary damage following ischemic insult. Nanozymes with robust anti-oxidative stress properties possess therapeutic possibility for ischemic insult. However, insufficiency of nanozyme accumulation in the neuronal mitochondria hindered their application. Herein, we constructed polydopamine-coated Prussian blue nanoparticles (PB@PDA NPs) to realize the targeting neuronal mitochondria for ischemic stroke, with the properties of antioxidant and anti-inflammation. After administration, much higher accumulation of PB@PDA NPs in the brain was observed compared to that in the PB NP group. Moreover, PB@PDA NPs effectively attenuated brain infarct than that of PB NPs in neonatal mice following hypoxia–ischemia (HI) insult. PB@PDA NPs mainly colocated with neuronal mitochondria in vivo and in vitro. Apart from attenuating oxidative stress, PB@PDA NPs also suppressed neuronal apoptosis and counteracted inflammation, which effectively promote a short- and long-term functional recovery in HI mice. Further, the therapeutic efficacy of PB@PDA NPs was also found in adult ischemic mice via tail vein injection. Collectively, these findings illustrate that PB@PDA NPs via system injection accumulate in neuronal mitochondria and are beneficial for ischemic stroke.

## Introduction

Stroke is one of the most common causes of death and the leading cause of disability in the infants and adulthood [1]. Among all types of strokes, brain ischemic stroke represents 87% of all cases, and neurological deficits such as motor impairment and inability to read or even aphasia are common consequences. Effective approaches for neurological functional recovery and long-term survival rate improvement after stroke are still urgently needed [2].

Strong evidence indicates that oxidative stress, caused by an imbalance between oxidants and antioxidants, is a major initiator and propagator of neuronal dysfunction and death and a key deleterious factor in cerebral ischemia. Reactive oxygen species (ROS) can be produced from normal metabolic processes in the living cells [3,4]. However, widespread overproductions of ROS triggered by ischemic insult modify or destroy cellular macromolecules directly, lead to inflammatory reactions and protease secretion, and damage cerebral tissues through various ways [4]. Increased ROS production is mostly from oxidative phosphorylation in the mitochondria [5]. Mitochondrial dysfunction is often related to oxidative stress pathologies. Thus, targeting mitochondria and ROS down-regulating treatment can maintain the mitochondrial integrity and a steady redox stature of cells will be of great therapeutic importance in cerebral ischemia [6]. To reduce mitochondrial ROS (mtROS) production, mitochondria-targeted antioxidants can specifically accumulate in mitochondria by affiliating to a lipophilic penetrating cation and prevent mitochondria from oxidative damage.

Currently, to protect ischemia-injured neurons, special attention has been devoted to developing feasible nanomaterials with enzyme-like activity (nanozymes), including CeO_2_, Fe_3_O_4_, Mn_3_O_4_, and V_2_O_5_, which present stable enzyme-like activity, robust antioxidative activity, and great physiological stability and have a low synthetic cost. Prussian blue (PB) nanoparticles (NPs), which present excellent biosafety, are a Food and Drug Administration (FDA)-approved antidote for cesium and thallium intoxication. As a biomimetic enzyme, PB NPs possess similar catalase (CAT)-, superoxide dismutase (SOD)-, and peroxidase (POD)-like activities. Recently, PB NPs have been applied to various central neural system disease with excessive oxidative stress, such as Parkinson’s disease [7], ischemia–reperfusion injured skin flap [8], and stroke [9]. Although PB is regarded as a classical drug in clinical therapy, the preparation of NPs due to its inorganic properties may have some problems such as poor stability in vivo, easy aggregation, and poor immunocompatibility [10]. Therefore, it is essential to find effective surface modification molecules to solve these problems. Polydopamine (PDA) has been widely used in surface modification of various inorganic or organic materials due to its unique properties, especially in the field of biomedicine [11]. Previous studies showed that the biocompatibility and hydrophilicity of the materials were decreased after PDA modification. Enhanced hydrophilicity can promote cell adhesion and other cell behaviors [12]. At the same time, because of the simple and easy modification of PDA, the material is easy to be favored [13]. Reports show that due to its unique properties, PDA can promote the adhesion, proliferation, and differentiation of nerve cells, thereby supporting and promoting the repair of peripheral nerve injuries [11].

Herein, we utilize a PDA-loaded PB NPs (PB@PDA NPs) to achieve stable solid nanostructures of PB NPs with high crystallinity. PDA with excellent photothermal effect can enhance antioxidant activity and biocompatibility of PB NPs during this process. The active targeting delivery of PB@PDA NPs to the ischemic brain was achieved by binding to neuronal mitochondria . The accumulated PB@PDA NPs in the damaged brain produced long-term therapeutic efficacy against ischemic stroke. Furthermore, we studied the detailed mechanism of PB@PDA NP treatment on ischemic stroke, including decreased neurons apoptosis, microglia activation, and ROS overproduction. This strategy will provide an applicative perspective for nanozyme therapy in various brain diseases.

## Materials and Methods

### Synthesis of PB NPs

PB NPs must be formed before synthesizing PB@PDA NPs. A typical method was used to prepare PB NPs according to a procedure described in a previous report [14]. In brief, potassium ferricyanide (131.7 mg) and polyvinylpyrrolidone (PVP, 3 g) were added to 40 ml of HCl solution (10 mM) under vigorous stirring at room temperature for 30 min. The mixture was then put into an electric oven and heated at 80 °C for 20 h for aging. Subsequently, the precipitates were collected by centrifugation and re-washed in distilled ethanol and water several times. After a further drying process, PB NPs were obtained and stored at room temperature for further use.

### Synthesis of PDA NPs

PDA NPs were prepared as control. Typically, 2 ml of NH_4_OH, 40 ml of ethanol, and 90 ml of water were first mixed under vigorous stirring at room temperature for 30 min. DA·HCl (500 mg) dissolved in water (10 ml) was then dropwise added to the above mixture under stirring for 24 h. After centrifuging, washing, and drying, PDA NPs were obtained and stored at room temperature for further use.

### Preparation of PB@PDA NPs

PB@PDA NPs were prepared by a one-step method. Before the preparation, 20 mg of PB NPs was first evenly dispersed in 40 ml of tris-HCl (10 mM, pH 8.5) through ultrasonic vibration. Dopamine hydrochloride (4 mg) was added into the PB solution under vigorous stirring at 600 rpm (attention to air circulation). After 1 h, PB@PDA NPs were obtained after full centrifugal cleaning.

### Characterization techniques

Transmission electron microscopy (TEM) imaging was executed using a FEI TalosF200x transmission electron microscope with field emission (Hillsboro, USA) at an operating voltage of 200 kV for PB NPs, PDA, and PB@PDA NPs to observe their sizes and morphologies, and each sample dispersed in water was dropped onto a carbon-coated copper grid and air dried before measurements. Fourier transform infrared (FTIR) spectra were recorded on a Nicolet 6700 FTIR spectrophotometer (Thermo Electron Corporation, Madison, WI). Samples were mixed with milled KBr crystals and pressed to form 13-mm-diameter disks before measurements. Thermogravimetric analysis (TGA) was executed using a Mettler Toledo TGA/DSC 3+ (Switzerland, Zurich). Each sample was prepared of 10 mg for testing in the range of 30 to 1,000 °C. Zeta potential and dynamic light scattering (DLS) measurements were performed using a Malvern Zetasizer Nano ZS model ZEN3600 (Worcestershire, UK) coupled with a standard 633-nm laser. Samples were dispersed in phosphate-buffered solution (PBS) with a concentration of 1 mg/ml before measurements. Ultraviolet–visible (UV–vis) spectra were collected using a Lambda 25 UV–vis spectrophotometer (Perkin Elmer, Waltham, MA). All samples were dispersed in water before measurements. Fluorescence spectra were recorded on a QuantMaster-40 fluorescence spectrophotometer (Protein Technologies Inc., Tucson, AZ). Atomic force microscopy (AFM) images of PB@PDA NPs were captured by a Dimension ICON instrument (Bruker, Massachusetts, USA). All samples were dispersed in water before measurements.

### Statistical analysis

All data are presented as mean ± standard deviation (SD) and are the result of 3 independent experiments, each performed at least in triplicate. The Shapiro–Wilk test was used to test whether the data conformed to the normal distribution, and equality of variance was confirmed using the *F* test. Outliers in normally distributed data sets were identified using Grubbs’ test and excluded from further analysis. If the data conformed to the normal distribution, the independent samples 2-tailed Student’s *t* test was used to compare the 2 groups, and the one-way analysis of variance (ANOVA) followed by post hoc Bonferroni (homogeneity of variances) was used to compare the multiple groups. However, if the assumption of homogeneity of variances or normality was not met, the one-way ANOVA followed by post hoc Tamhane T2 was utilized. If it did not conform to the normal distribution, the Mann–Whitney *U* test was used between the 2 groups, and the Kruskal–Walli’s test was used between multiple groups. A repeated-measures 2-way ANOVA analysis was performed to analyze the body weight, average fluorescence intensity of animal image, and number of microglia intersections. The survival rate of mice after HI in different groups was analyzed using the chi-square test. Pearson correlation test was used to evaluate correlations between brain damage and PB@PDA NP concentration. Normally distributed data are presented as bar plots. Nonnormally distributed data are presented as box plots. The longest branch length and cell body area of microglia data in Iba-1 immunohistochemical staining are presented as violin plot. The statistical differences are presented at probability levels of *P* < 0.05 (*), *P* < 0.01 (**), and *P* < 0.001 (***). Calculations were performed with standard statistical software (GraphPad Prism 8.0.1, GraphPad Software, La Jolla, USA).

## Results

### Synthesis and characterization of the PB@PDA NPs

**Fig. 1.**
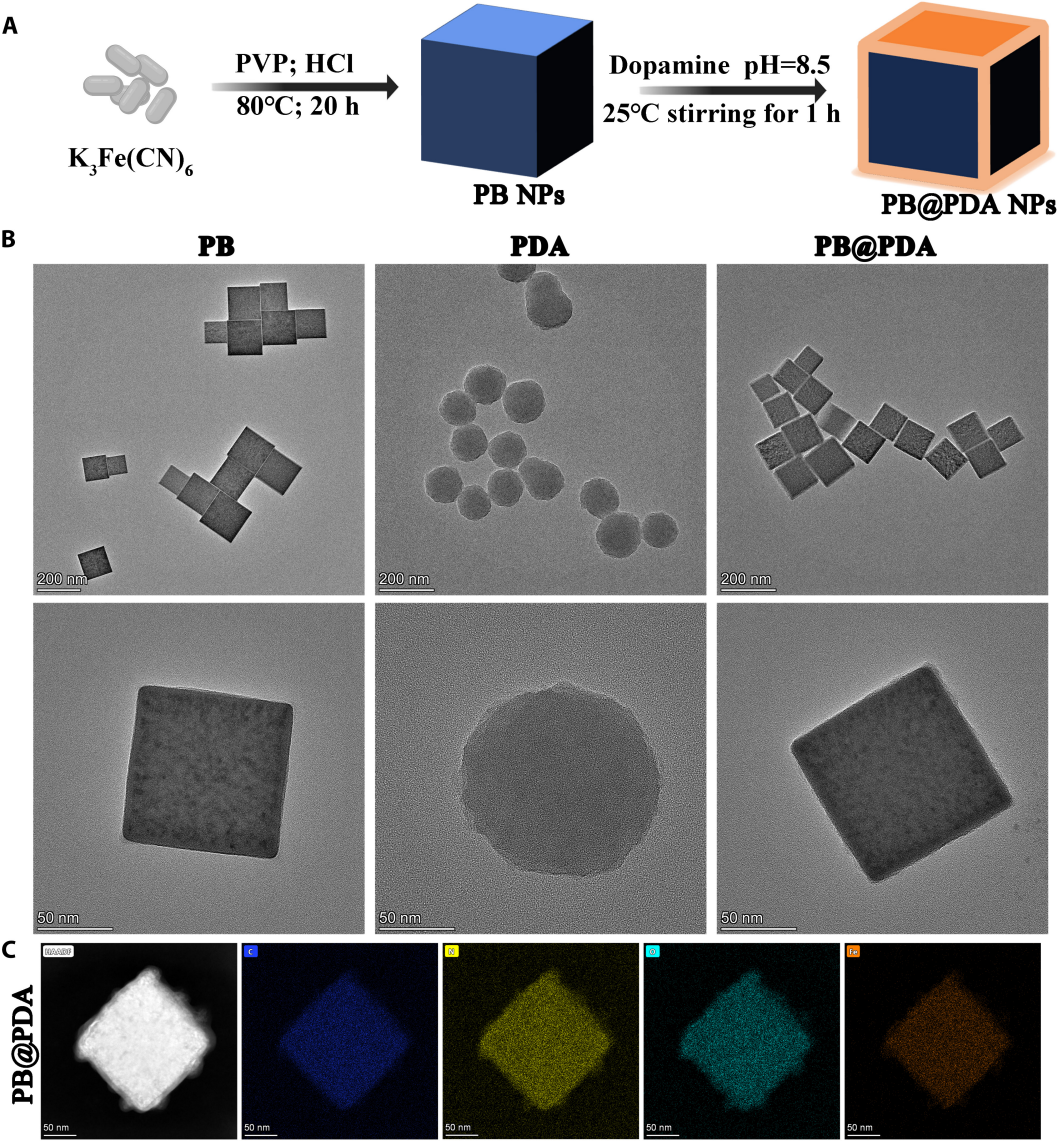
Preparation and synthesis of the PB@PDA NPs. (A) Diagram of PB@PDA NP possible formation mechanism. (B) Representative TEM images of PB NPs, PDA NPs, and PB@PDA NPs. (C) Elemental mapping spectrum of PB@PDA NPs, which indicated that C (blue), N (yellow), O (cyan), and Fe (orange) were found in the synthesized nanomaterials.

In this work, the PB@PDA NPs were constructed via a two-step method for brain trauma therapy (Fig. 1A). TEM was used to characterize the morphology and size of every product. Each NPs possess relatively uniform shapes and sizes. The average size of PB, PDA, and PB@PDA NPs in the TEM image is 130 ± 30 nm, 140 ± 20 nm, and 115 ± 14 nm, respectively (Fig. 1B). At the elemental level, the elemental mapping demonstrated the composition of the main components of the hollow structure and the distribution of Fe, C, N, and O elements (Fig. 1C). Subsequently, the hydrodynamic sizes, polymer dispersity index (PDI), and zeta potentials of each NPs were measured (Table S1). The particle size of PB, PDA, and PB@PDA NPs is 291.2 ± 13.2 nm, 680.1 ± 8.9 nm, and 485.1 ± 10.8 nm, respectively, which are larger than those observed by TEM due to the existence of hydration layer. PB NPs are not alkali resistant, and the polymerization of PDA NPs is in an alkaline environment (pH 8.5), so the hydrodynamic sizes of PB@PDA NPs are larger than PB NPs (Fig. S1). PDI of both PB and PB@PDA NPs shows that they can form stable dispersed solutions and have stable colloidal dispersion. Moreover, the different zeta potentials of these 3 NPs indirectly show that PDA is successfully wrapped on the surface of PB NPs.

**Fig. 2.**
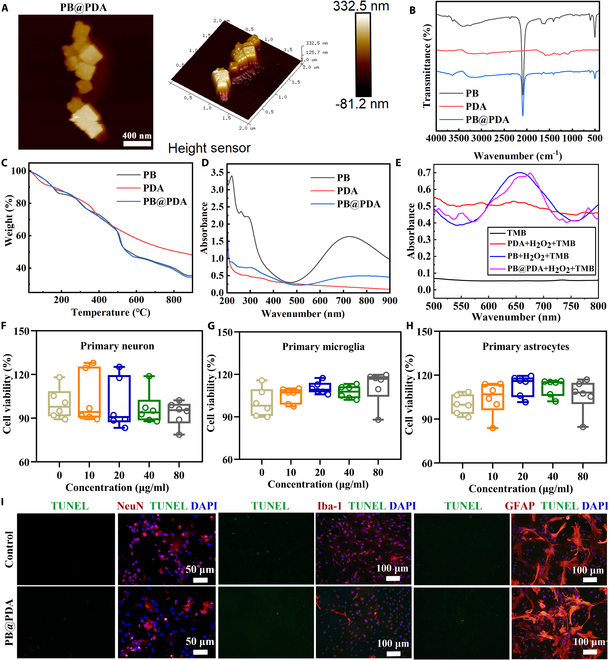
Characterization of the PB@PDA NPs. (A) Representative 2D and 3D AFM images of PB@PDA NPs. (B) FTIR spectra of PB, PDA, and PB@PDA NPs. (C) TGA of PB, PDA, and PB@PDA NPs. (D) The UV–vis absorption spectra of PB, PDA, and PB@PDA NPs. (E) UV–vis spectra of NPs after treatment with H_2_O_2_ and TMB. (F) Cytotoxicity of primary neuron after incubation with different concentrations of PB@PDA for 24 h (data are presented as means ± SD of 6 per group). (G) Cytotoxicity of primary microglia after incubation with different concentrations of PB@PDA for 24 h (data are presented as means ± SD of 6 per group). (H) Cytotoxicity of primary astrocytes after incubation with different concentrations of PB@PDA for 24 h (data are presented as means ± SD of 6 per group). (I) Representative immunofluorescence images of NeuN (red) and TUNEL staining (green) in primary neuron. Scale bar, 50 μm. Representative immunofluorescence images of Iba-1 (red) and TUNEL staining (green) in primary microglia. Scale bar, 100 μm. Representative immunofluorescence images of GFAP (red) and TUNEL staining (green) in primary astrocytes. Scale bar, 100 μm.

Representative AFM images showed that the PB@PDA NPs were nearly square, and the height sensor of the particle is about 332.5 nm (Fig. 2A). Next, the results of FTIR spectra and TGA offered additional demonstration of the successful preparation of PB@PDA NPs. As shown in Fig. 2B, the intense absorption peaks around 2,080, 590, and 1,656 cm^−1^ are discovered in PB NPs and PB@PDA NPs owing to the telescopic vibration of C≡N bond, Fe–CN bond, and C═O bond. The absorption band between 3,500 and 3,200 cm^−1^ is found in both PDA NPs and PB@PDA NPs due to the stretching vibration of O–H, N–H, and NH_2_. In addition, according to the change of sample quality recorded by thermogravimetric analyzer in this heating interval (Fig. 2C), the results of TGA display that PB and PB@PDA NPs have similar weightlessness trends around both 151 and 510 °C, while PDA and PB@PDA NPs have similar weightlessness trends around 375 °C. TGA possesses similar conclusion, also suggesting that the successful preparation of PB@PDA NPs.

The UV–vis absorption spectra of PB, PDA, and PB@PDA NPs are shown in Fig. 2D. The obtained PB NPs possess a broad absorption from 500 to 900 nm with a strong absorption peak, while the absorbance peak from 500 to 900 nm of PB@PDA NPs decreased slightly due to the surface modification by PDA. Subsequently, the POD-like activity of PB, PDA, and PB@PDA NPs was estimated by tetramethylbenzidine (TMB) chromogenic experiment. As shown in Fig. 2E, the increase in absorbance at 650 nm for PB and PB@PDA NPs in the presence of H_2_O_2_ suggests the occurrence of oxidizing reaction of TMB.

Encouraged by the catalytic activity of PB@PDA observed in the above assays, we next investigated whether PB@PDA could exert cytotoxicity effects in cells under different conditions. Primary neuron, microglia, and astrocytes were used to evaluate the cytotoxicity effects of PB@PDA NPs on neuronal and glia cells, respectively. The PB@PDA NPs with concentrations of 10, 20, 40, and 80 μg/ml exerted no obvious cytotoxicity to mouse primary neuron, microglia, and astrocytes (Fig. 2F to I). These results indicate the biocompatibility of PB@PDA NPs, as it does not exhibit any adverse effects on cells.

### PB@PDA NPs protected against hypoxia–ischemia-induced neuronal damage in the early phase in neonatal mice

**Fig. 3.**
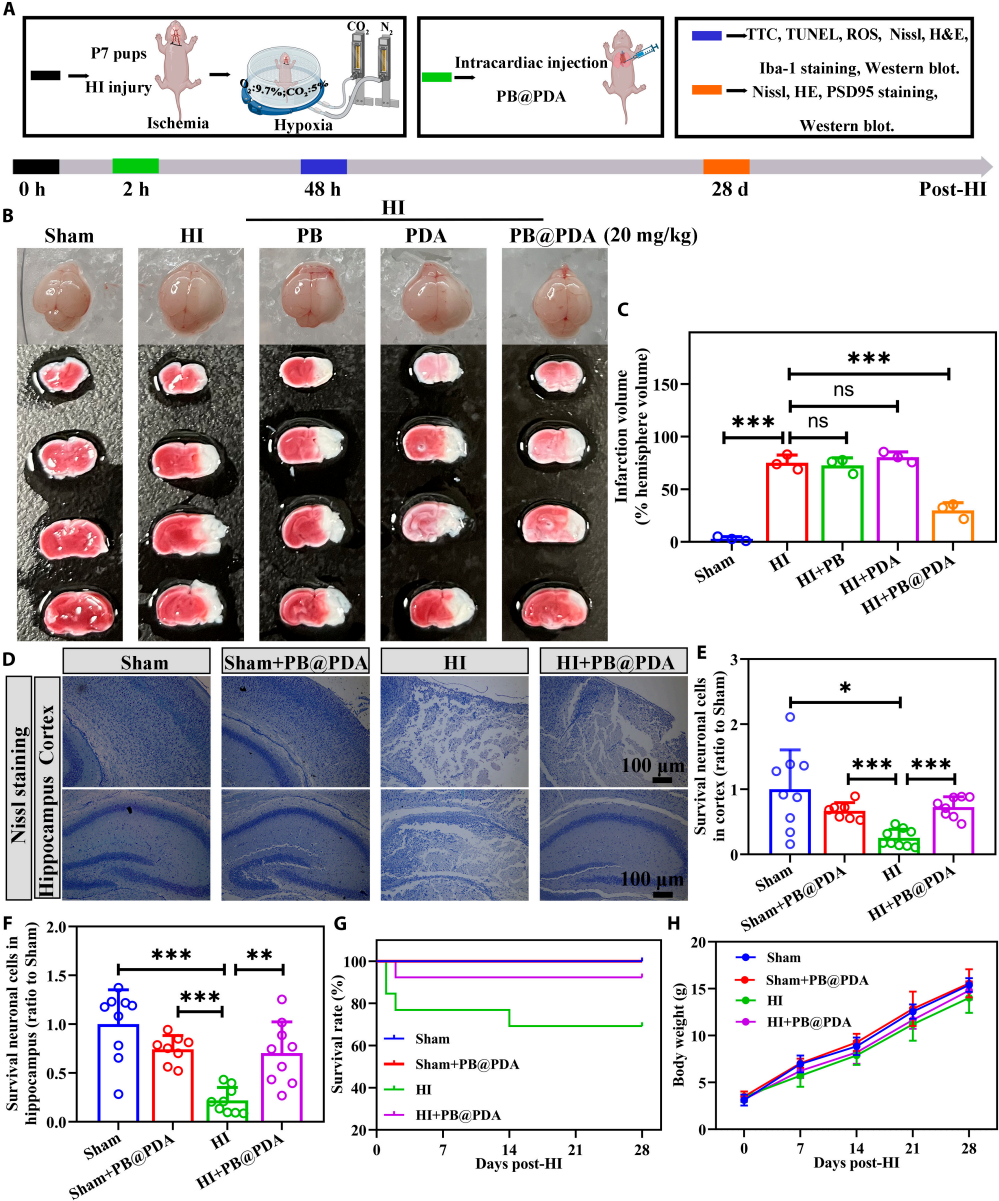
Protective effect and biosafety of PB@PDA NPs on HI injury model. (A) Treatment schedule. PB@PDA NPs (20 mg/kg) was intravenously injected intracardially into mice at 2 h after HI. Biological experiments were conducted at 48 h and 28 d after HI. (B) Representative samples stained with TTC at 48 h following HI insult under PB, PDA, and PB@PDA NP treatment. (C) Infarct volumes were calculated as the percentage of infarct volume to hemisphere brain (data are presented as means ± SD of 3 per group, one-way ANOVA, ns: not significant, ***P < 0.001). (D) Representative Nissl staining images in cortex and hippocampus 48 h after HI. Scale bar, 100 μm. (E) Nissl bodies were counted and analyzed in the cortex (data are presented as means ± SD of 7 to 9 per group, one-way ANOVA followed by post hoc Tamhane T2, *P < 0.05, ***P < 0.001). (F) Nissl bodies were counted and analyzed in the hippocampus (data are presented as means ± SD of 7 to 9 per group, one-way ANOVA followed by post hoc Tamhane T2, **P < 0.01, ***P < 0.001). (G) Survival rate at 0 to 28 d after HI. (H) Changes in body weights of test mice observed in different treatment groups (data are presented as means ± SD of 6 per group).

Next, we explored the therapeutic efficacy of PB@PDA NPs in the neonatal hypoxia–ischemia (HI) model. Neonatal HI brain damage is a common brain injury caused by hypoxia and ischemia in perinatal period, and is one of the most used models to simulate stroke in neonatal mice [15]. PB@PDA NPs were administered at 2 h after HI injury via intracardial injection (Fig. 3A). Before the application of PB@PDA NPs in brain injury treatment, their hemolysis rates were evaluated. Blood compatibility is a crucial consideration for the development of materials for blood-contacting devices [16]. Compared to the positive control (1% Triton X-100), the hemolysis rates of red blood cells treated with PB@PDA NPs under the concentration of 1, 2, and 3 mg/ml were less than 5%, suggesting the good hemocompatibility of PB@PDA NPs within the dose range (Fig. S2A). The doses of PB@PDA NPs were selected based on data from our pilot study (comparing 1, 5, 10, 20, and 40 mg/kg; which found that 20 mg/kg PB@PDA had the effect on infarction areas; Fig. S2B to D). In a certain concentration range, the brain infarct volume and PB@PDA NP concentration were dose dependent (Fig. S2E). As shown in Fig. 3B and C, PB@PDA NP treatment (20 mg/ml) reduced HI-induced brain edema within the ipsilateral side as compared with the HI group (*P* < 0.001), while PB NP treatment at 20 mg/ml and PDA NP treatment at 20 mg/ml had no effect on edema and brain infarction after HI brain injury. Nissl staining showed that neuronal cells in the HI group were loosely arranged or missing, and Nissl bodies were lightly stained or even dissolved compared with that in Sham and HI + PB@PDA groups. The number of survival cells of ipsilateral cortex (*P* < 0.001, versus HI) and hippocampus (*P* < 0.01, versus HI) was marked increased in the HI + PB@PDA group, indicating PB@PDA NPs reversing HI-induced neuronal injury (Fig. 3D to F).

We examined the mortality of each group recorded daily for 35-d period. Mice in the Sham and Sham + PB@PDA groups showed 100% survival within the whole observation period. All the deaths occurred within the first 14 d of HI, and the overall survival ratio of the PB@PDA NP treatment group was 92.3%, much higher than 69.2% of PBS control, suggesting the excellent capability of PB@PDA NPs on reducing mortality (Fig. 3G). Mice in Sham and HI groups were observed for 28 consecutive days following intracardial administration since signs of acute toxicity were exhibited through changes in physical appearance and behavior. There is similar birth weight among Sham, Sham + PB@PDA, HI, and HI + PB@PDA (Fig. 3H). Moreover, Sham mice receiving PB@PDA NPs showed no neuronal injury or lesions throughout the brain tissue. No significant difference was detected between the untreated Sham (Sham) and PB@PDA NP-treated Sham group (Sham + PB@PDA) in the survival and brain morphology (Fig. S3); therefore, the PB@PDA NP-treated Sham group was not further examined.

Next, the major organs were sectioned and stained with hematoxylin and eosin (H&E) for histological examination to evaluate the long-term potential toxicity for peripheral organs, at 28 d following PB@PDA nanozyme administration. As shown in Fig. S4, cardiomyocyte, hepatocytes, and nephrocytes appeared normal, and there were no inflammatory infiltrates. Spleen, lung, and kidney have no pathological injuries. These data suggested that the nanozymes had no toxicity in the brain and peripheral organs. Importantly, biochemistry assays of plasma revealed no significant differences between the control and PB@PDA NP treatment groups, further confirming the biosafety of PB@PDA in vivo (Fig. S5). Taken together, these results demonstrated that PB@PDA NPs could attenuate neuronal damage in mice after HI injury.

### PB@PDA NPs reached the brain and colocalized in the mitochondria of neuron

**Fig. 4.**
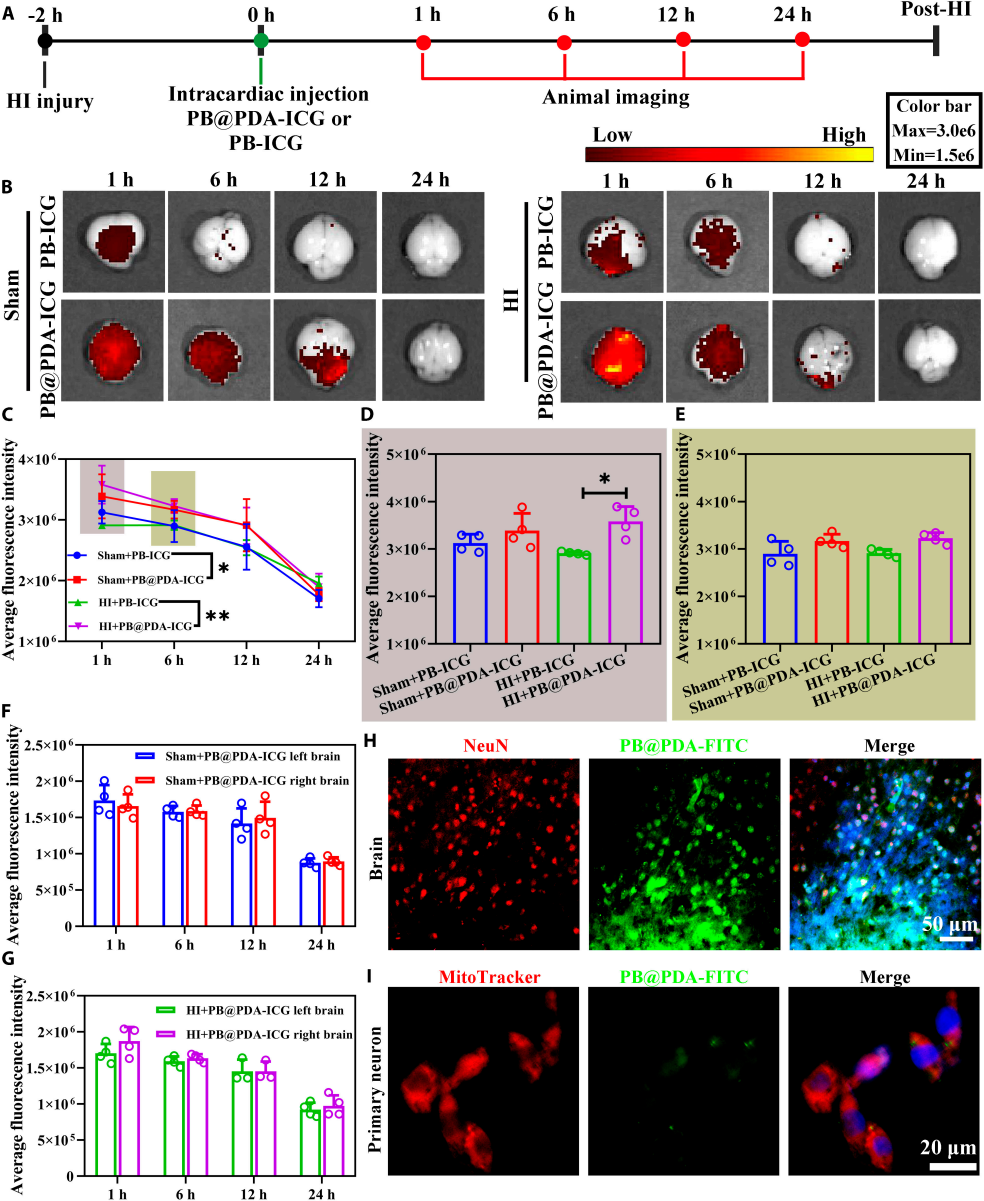
Ex vivo biodistribution of therapeutic PB@PDA NPs. (A) Time axis of animal imaging in the HI model. (B) Ex vivo fluorescence images of brain tissue from mice at 1, 6, 12, and 24 h after intracardiac injection with ICG-labeled PB NPs or ICG-labeled PB@PDA NPs. (C) The fluorescence level of the brain was quantified by the mean fluorescence intensity (data are presented as means ± SD of 3 to 4 per group, 2-way ANOVA followed by post hoc Bonferroni, *P < 0.05, **P < 0.01). (D) The fluorescence level of the brain was quantified by the mean fluorescence intensity 1 h after intracardiac PB@PDA NP injection (data are presented as means ± SD of 4 per group, one-way ANOVA followed by post hoc Bonferroni, *P < 0.05). (E) The fluorescence level of the brain was quantified by the mean fluorescence intensity 6 h after intracardiac PB@PDA NP injection (data are presented as means ± SD of 4 per group). (F) The fluorescence level of the left and right brain was quantified by the mean fluorescence intensity in the Sham group after intracardiac PB@PDA NP injection (data are presented as means ± SD of 4 per group). (G) The fluorescence level of the left and right brain was quantified by the mean fluorescence intensity in the HI group after intracardiac PB@PDA NP injection (data are presented as means ± SD of 3 to 4 per group). (H) Representative fluorescence staining of NeuN of brain section 1 h after intracardiac injection of FITC-labeled PB@PDA NPs at 1 h after HI. 4′,6-Diamidino-2-phenylindole (DAPI) (blue, nuclei), FITC (green, PB@PDA NPs), and NeuN (red, neuron). Scale bar, 50 μm. (I) Representative images on MitoTracker Red CMXRo in primary neuron after treatment with FITC-labeled PB@PDA NPs. DAPI (blue, nuclei), FITC (green, PB@PDA NPs), and MitoTracker (red, mitochondria). Scale bar, 20 μm.

To gain insight into the distribution of PB@PDA nanozyme in neonatal mice, we used the indocyanine green (ICG)-labeled PB@PDA NPs or PB NPs (PB@PDA-ICG NPs or PB-ICG NPs; Fig. S6), which were administered at 1 h after HI injury. The distribution of PB@PDA-ICG NPs and PB-ICG NPs was investigated at 1, 6, 12, and 24 h after administration in various mouse ex vivo tissues, including brain, heart, liver, kidney, lung, and spleen with in vivo imaging system (IVIS) imaging system (Fig. 4A). PB-ICG NPs were hardly enriched in the brains of Sham and HI groups after PB-ICG NP administration, while PB@PDA-ICG NPs remarkably accumulated in Sham (*P* < 0.05, versus Sham + PB-ICG NPs) and HI (*P* < 0.01, versus HI + PB-ICG NPs) brains after PB@PDA-ICG NP administration (Fig. 4B and C). The average fluorescence intensity of PB@PDA-ICG NPs was higher than that of PB-ICG NPs in the HI group at 1 h (*P* < 0.05) and 6 h after administration (Fig. 4D and E). In the Sham group, the ICG fluorescence signal of PB@PDA-ICG NPs was similar between the ipsilateral and contralateral hemispheres (Fig. 4F). However, in the HI group, the ICG fluorescence signal of the ipsilateral hemisphere was stronger than that of the contralateral hemisphere, although there was no statistical difference (Fig. 4G). Moreover, significant decreases in ICG fluorescence signal in the brain were observed in each group over the period from 1, 6, 12, to 24 h, suggesting that each group showed similar reduction in ICG fluorescence signal over time. In addition, PB@PDA-ICG NPs and PB-ICG NPs were found in the kidney, liver, lung, heart, and spleen regardless of Sham and HI groups, and the fluorescence signal decreased with the increase of time. In addition, we compared the distribution of PB@PDA-ICG NPs in the brain and organs, and the results showed that the content of PB@PDA-ICG NPs in the liver was the highest after intracardiac injection of PB@PDA-ICG NPs for 1, 6, and 12 h, followed by the brain. The PB@PDA-ICG NP content is highest in the brain tissue for 24 h (Figs. S7 to S10). The efficiency of PB@PDA-ICG NP delivery to the brain is about 20 to 30%, and the rest is distributed in various organs after intracardiac injection of PB@PDA-ICG. PB@PDA-ICG NPs via intracardiac injection can reach the brain at 1 h and last for 24 h after administration (Fig. S11), while the accumulation of PB@PDA-ICG NPs in other organs significantly decreased at 24 h after administration, suggesting good biological stability of PB@PDA-ICG NPs in the brain.

To investigate the cellular distribution of PB@PDA NPs, immunofluorescence staining of fluorescein isothiocyanate-labeled PB@PDA NPs (PB@PDA-FITC NPs) with specific markers for neurons (NeuN), microglia (Iba-1), and astrocyte [glial fibrillary acidic protein (GFAP)] was performed at 1 h post-HI injury after administration. PB@PDA-FITC NPs mainly colocalized in NeuN^+^ neuronal cells (Fig. 4H), while little immunofluorescence of PB@PDA-FITC NPs was seen within GFAP^+^ astrocytes and Iba-1^+^ microglia (Fig. S12) at 1 h after HI. Moreover, we also found that PB@PDA-FITC NPs (green fluorescence) mainly incorporated into primary neurons and easily colocalized with mitochondria of neurons (MitoTracker, red fluorescence) at 2 h after administration (Fig. 4I). In addition, we found that PB@PDA-FITC NPs can also colocate with mitochondria in AML12 cell (a mouse liver cell line) (Fig. S13). In summary, PB@PDA NPs can target the damaged brain area and colocate with the mitochondria of neurons to play an antioxidant role after intracardiac administration.

### PB@PDA NPs increased oxidative defenses following HI insult in neonatal mice

**Fig. 5.**
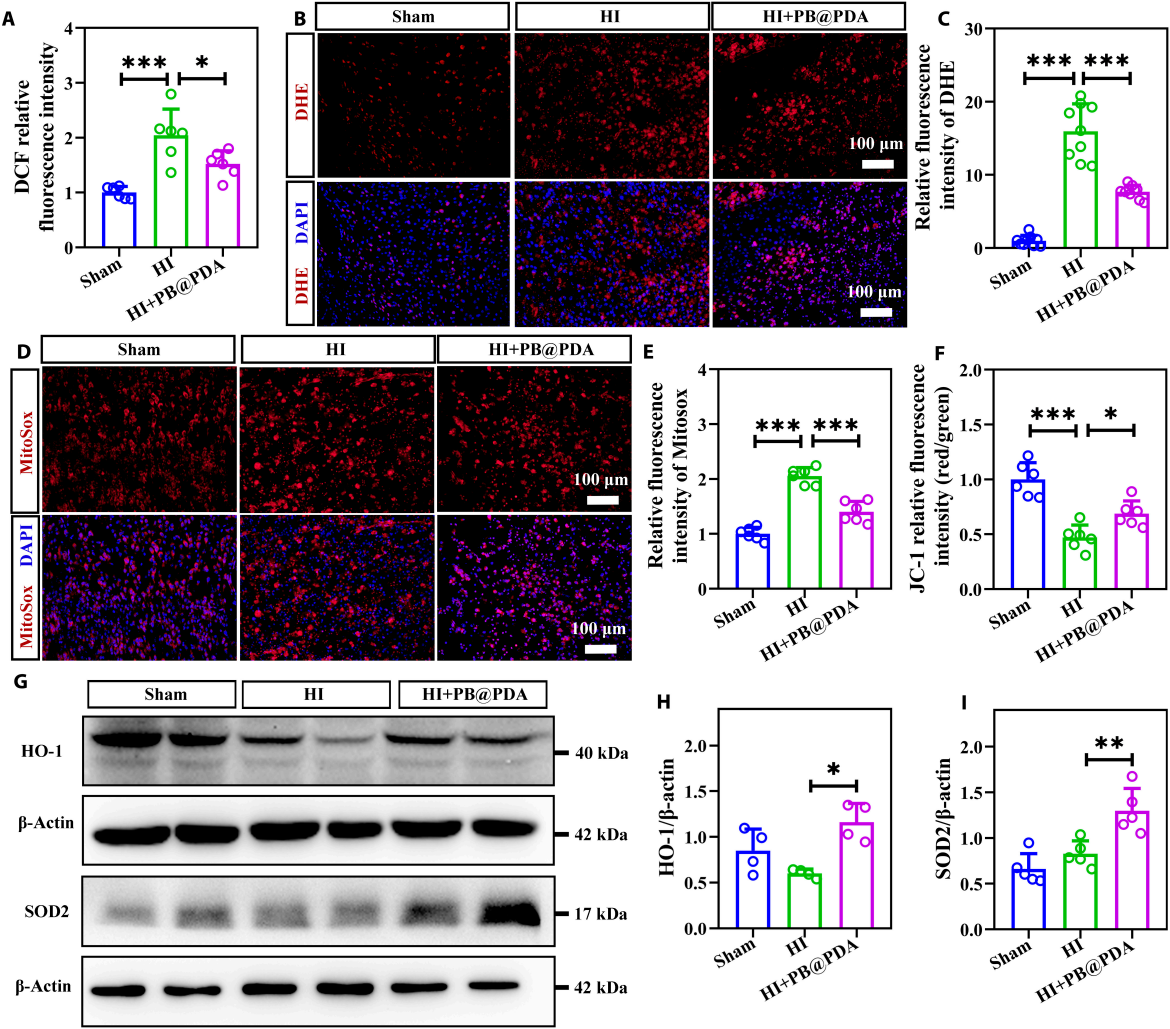
PB@PDA NPs improved oxidative stress and mitochondrial function following HI injury. (A) The intensity of ROS in cell suspension of brain tissue after different treatments was measured at 48 h after HI by fluorescence microplate (data are presented as means ± SD of 6 per group, one-way ANOVA followed by post hoc Bonferroni, *P < 0.05, ***P < 0.001). (B) Representative fluorescence staining micrographs for ROS in the cortex of each group at 48 h after HI. DAPI (blue, nuclei) and DHE (red, ROS). Scale bar, 100 μm. (C) Quantification of DHE fluorescence intensity on frozen sections for the detection of intracellular ROS (data are presented as means ± SD of 9 per group, one-way ANOVA followed by post hoc Tamhane T2, ***P < 0.001). (D) Representative MitoSox Red staining images for the detection of mtROS after different treatments. Scale bar, 100 μm. (E) The intensity of MitoSox in cell suspension of brain tissue after different treatments was measured at 48 h after HI by fluorescence microplate (data are presented as means ± SD of 6 per group, one-way ANOVA followed by post hoc Bonferroni, ***P < 0.001). (F) The intensity of JC-1 aggregate/monomer ratio in cell suspension of brain tissue after different treatments was measured at 48 h after HI by fluorescence microplate for mitochondrial membrane potential (data are presented as means ± SD of 6 per group, one-way ANOVA followed by post hoc Bonferroni, *P < 0.05, ***P < 0.001). (G) Western blots showing the expression of cleaved HO-1 and SOD2 in mouse brain tissues at 48 h after HI. (H) Quantitative analysis on the expression of HO-1 in (G) (data are presented as means ± SD of 4 per group, one-way ANOVA followed by post hoc Tamhane T2, *P < 0.05). (I) Quantitative analysis on the expression of SOD2 in (G) (data are presented as means ± SD of 5 per group, one-way ANOVA followed by post hoc Bonferroni, **P < 0.01).

The ROS levels in the lysis of fresh brain tissues were probed by dichlorodihydrofluorescein (DCFH), whose intensity was positively and quantitatively correlated with ROS. Compared with the HI group, PB@PDA NPs led to a 25.6% reduction in DCFH-DA fluorescence density (*P* < 0.05) at 48 h following HI insult using a fluorescence microplate reader (Fig. 5A). In line with this observation, DHE staining also found that PB@PDA NPs led to a 51.9% reduction in dihydroethidium (DHE) fluorescence density (*P* < 0.001) (Fig. 5B and C).

Mitochondrial damage was inevitable after ischemia–reperfusion, so maintaining mitochondrial function is critical to reversing ischemic damage [17]. Therefore, we then examined the effects of PB@PDA NPs on mtROS and mitochondrial membrane potential. In line with this observation, mtROS level was detected by MitoSox staining. PB@PDA NP treatment efficiently decreased mtROS levels (*P* < 0.001) (Fig. 5D and E). Compared with the Sham group, HI led to a 53.0% reduction in mitochondrial membrane potential with a fluorescence microplate reader (*P* < 0.001). PB@PDA NP treatment efficiently increased mitochondrial membrane potential (*P* < 0.05; Fig. 5F). Consistently, PB@PDA NP treatment remarkedly increased the expression of antioxidative factors, including HO-1 (*P* < 0.05) and SOD2 (*P* < 0.01), in the ipsilateral cortex following HI insult (Fig. 5G to I). HO-1 and SOD2 as endogenous antioxidant enzymes in neurons could exert antioxidant action to remove ROS [18]. These data show that after HI injury, PB@PDA NPs could effectively reduce ROS levels and mitochondrial function.

### PB@PDA NPs inhibited neuronal apoptosis in the early phase of HI insult in neonatal mice

**Fig. 6.**
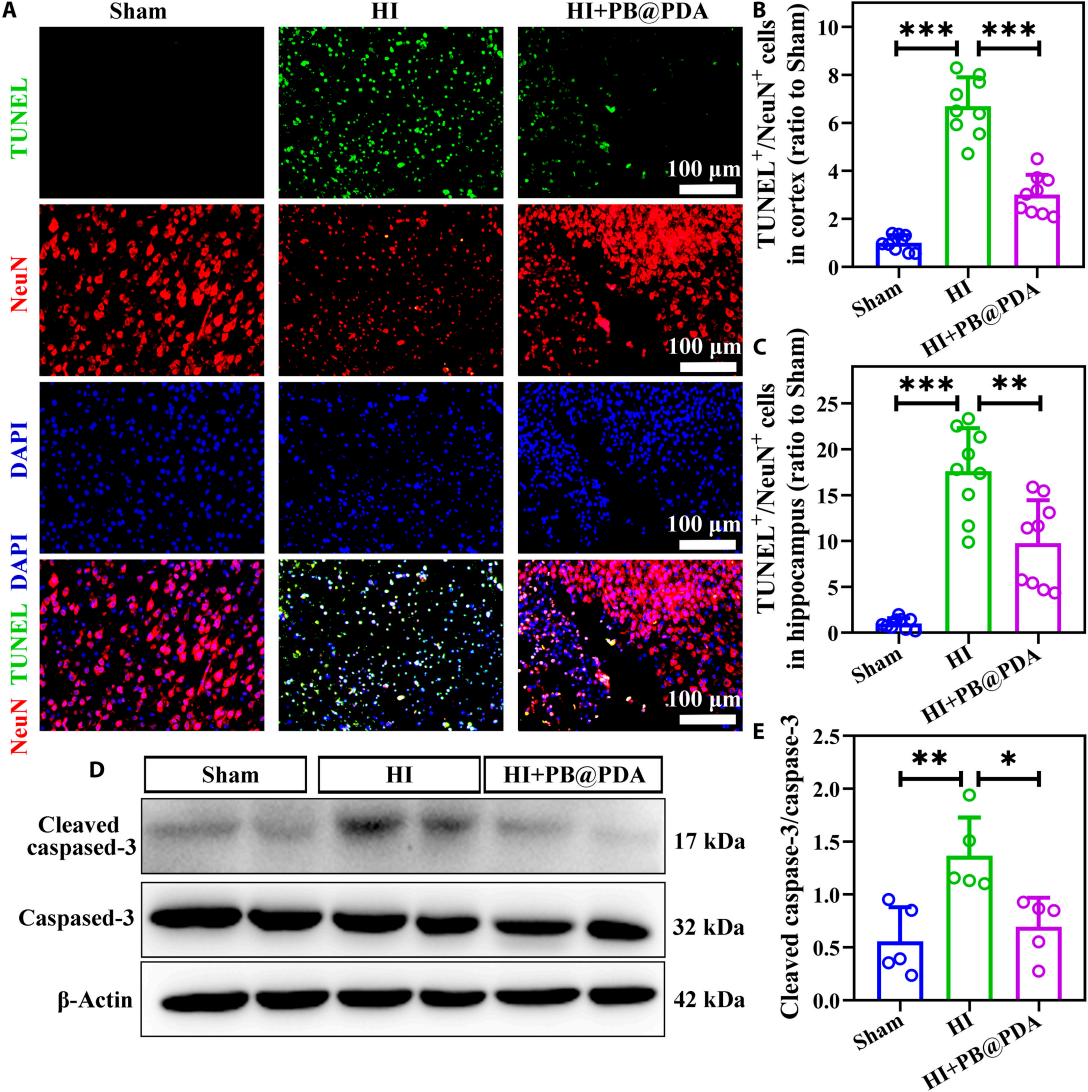
PB@PDA NPs reduce neuronal apoptosis following HI injury. (A) Representative immunofluorescence images of NeuN (red) and TUNEL staining (green) in mouse brain tissues at 48 h after HI. Scale bar, 100 μm. (B and C) TUNEL-positive cells were counted and analyzed in the cortex and hippocampus (data are presented as means ± SD of 9 per group, one-way ANOVA followed by post hoc Tamhane T2, **P < 0.01, ***P < 0.001). (D) Western blots showing the expression of cleaved caspase-3 and caspase-3 in mouse brain tissues at 48 h after HI. (E) Quantitative analysis on the expression of cleaved caspase-3 and caspase-3 in (D) (data are presented as means ± SD of 5 per group, one-way ANOVA followed by post hoc Bonferroni, *P < 0.05, **P < 0.01).

Previous studies have shown that cytochrome c is released into the cytoplasm due to the destruction of mitochondrial structure, leading to the activation of the apoptosome and caspase-mediated apoptosis after HI brain injury [19]. To fully understand the potential neuroprotection mechanisms, brain sections were collected at 48 h after HI. Neuronal survival was assessed by terminal deoxynucleotidyl transferase–mediated deoxyuridine triphosphate nick end labeling (TUNEL)/NeuN double-positive staining. Results revealed that PB@PDA NP treatment significantly promoted neuronal survival in the cortex (*P* < 0.001) and hippocampus (*P* < 0.01). PB@PDA NP treatment alleviated the infarct area and promoted neuronal survival in the cortex and hippocampus of HI mice (Fig. 6A to C); therefore, the hippocampus was not further examined (Fig. S14). When the body receives the apoptotic signal, caspase-3 is activated to cleaved caspase-3, which induces apoptosis. Following HI insult, levels of cleaved caspase-3 in the ipsilateral cortex were increased compared with those of the Sham group (*P* < 0.01), while cleaved caspase-3 was decreased in the PB@PDA NP treatment group (*P* < 0.05) as determined using Western blot analysis (Fig. 6D and E). Overall, PB@PDA NPs play a neuroprotective role by inhibiting caspase-3-mediated apoptosis signaling pathway after HI injury.

### PB@PDA NPs decreased glial activation and pro-inflammatory cytokine expression in the early phase in neonatal mice

**Fig. 7.**
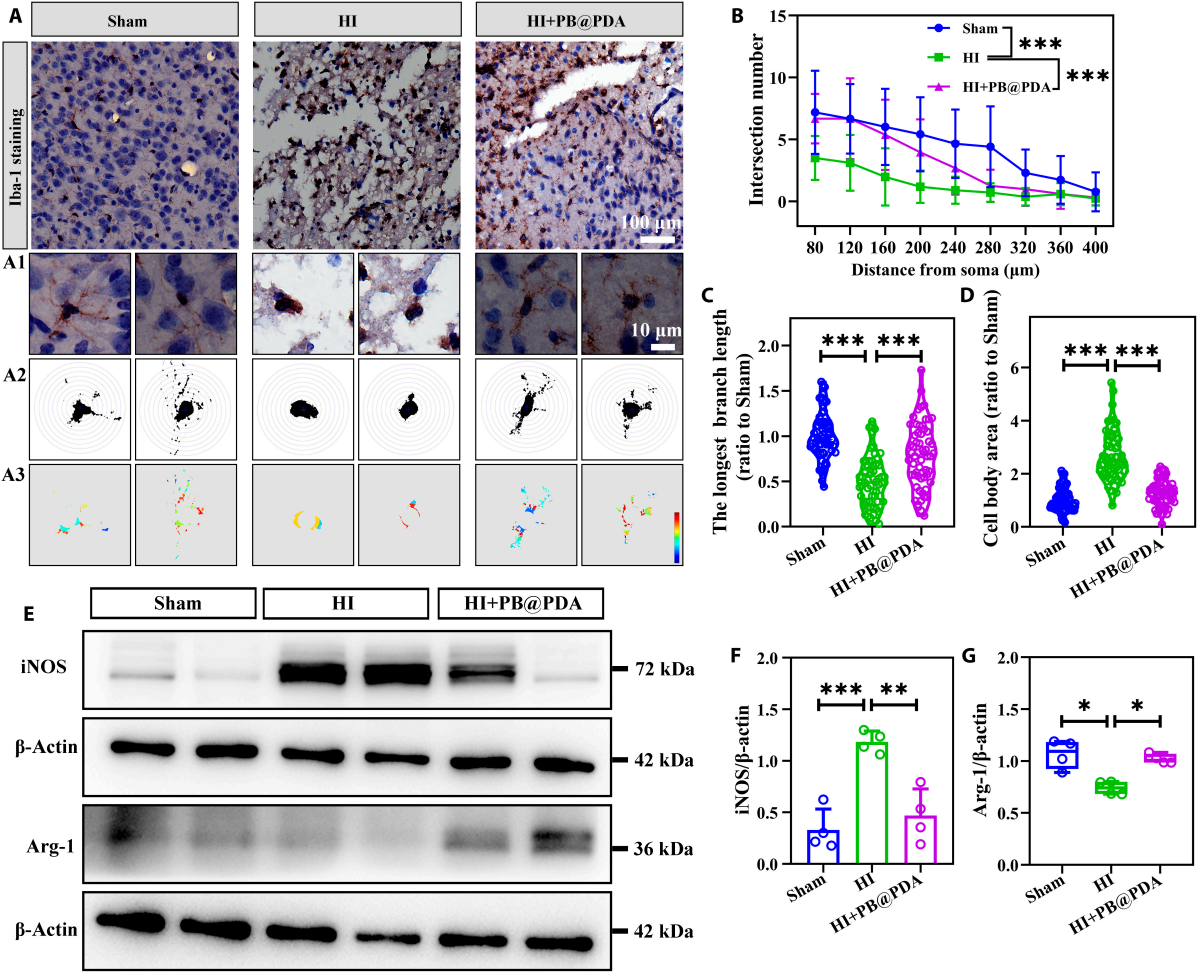
PB@PDA NPs reduce activated microglia and inflammatory cytokines following HI injury. (A) Immunohistochemical staining of Iba-1 in the cortex region of mouse brains from Sham or HI PB@PDA-treated mice. Scale bar, 100 μm. A1 is an enlarged view of microglia. Scale bar, 10 μm. A2 is the intersection diagram for Sholl analysis of microglia using ImageJ software. A3 is a pseudo-color picture of the intersections of microglia processes. (B) Quantitative analysis of number of intersections at different distances from soma in Iba-1-positive microglia (data are presented as means ± SD of 60 per group, 2-way ANOVA followed by post hoc Bonferroni, ***P < 0.001). (C) Quantitative analysis of the longest branch length in Iba-1-positive microglia (data are presented as means ± SD of 60 per group, one-way ANOVA followed by post hoc Tamhane T2, ***P < 0.001). (D) Quantitative analysis of the cell body area in Iba-1-positive microglia (data are presented as means ± SD of 60 per group, Kruskal–Wallis ANOVA test, ***P < 0.001). (E) Western blots showing the expression of cleaved inducible nitric oxide synthase (iNOS) and Arg-1 in mouse brain tissues at 48 h after HI. (F) Quantitative analysis on the expression of iNOS in (E) (data are presented as means ± SD of 4 per group, one-way ANOVA followed by post hoc Bonferroni, **P < 0.01, ***P < 0.001). (G) Quantitative analysis on the expression of Arg-1 in (E) (data are presented as means ± SD of 4 per group, Kruskal–Wallis ANOVA test, *P < 0.05).

We next examined the effect of PB@PDA NPs on neuroinflammation. Microglial cell activation was evaluated by studying the immunohistochemical changes in the expression of Iba-1 protein within the ipsilateral cortex 48 h after HI. The results of this assay showed that PB@PDA NP treatment significantly inhibited microglial activation by decreasing the number of Iba-1^+^ cells and score (*P* < 0.05) (Fig. S15). Microglia cells in Sham mice displayed a more stellate morphology, and cells found in HI mice appeared with bigger soma and thicker branches. HI insult decreased the number of endpoints (*P* < 0.001) (Fig. 7A and B), processed length of branches per cell (*P* < 0.001) (Fig. 7C), and increased bigger soma, while the above morphology improved significantly after PB@PDA NP treatment (Fig. 7D). In addition, PB@PDA NPs significantly decreased the pro-inflammatory cytokines inducible nitric oxide synthase (*P* < 0.01) and increased the anti-inflammatory cytokine arginase-1 (*P* < 0.05) in the ipsilateral cortex as compared to vehicle-treated HI mice (Fig. 7E to G). These results suggest that PB@PDA NPs alleviated microglia activation and neuroinflammation following HI insult.

### PB@PDA NP treatment alleviated synaptic damage following HI insult in neonatal mice

**Fig. 8.**
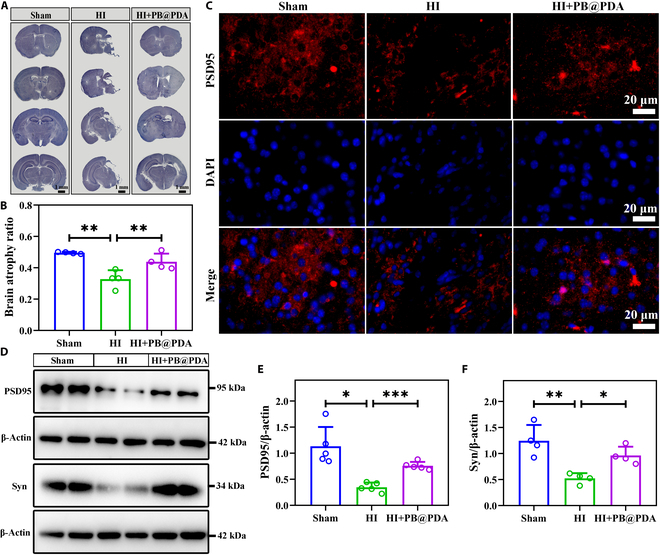
PB@PDA NPs improved synaptic function at 28 d following HI injury. (A) Representative Nissl staining images in different coronary sections of the brain at 28 d after HI. Scale bar, 1 mm. (B) Quantification of cerebral atrophy at 28 d after HI (data are presented as means ± SD of 4 per group, one-way ANOVA followed by post hoc Bonferroni, **P < 0.01). (C) Representative immunofluorescence images of PSD95 (red) and DAPI staining (blue). (D) Western blots showing the expression of cleaved HO-1 and SOD2 in mouse brain tissues at 48 h after HI. (E) Quantitative analysis on the expression of PSD95 in (D) (data are presented as means ± SD of 5 per group, one-way ANOVA followed by post hoc Tamhane T2, *P < 0.05, ***P < 0.001). (F) Quantitative analysis on the expression of Syn in (D) (data are presented as means ± SD of 4 per group, one-way ANOVA followed by post hoc Bonferroni, *P < 0.05, **P < 0.01).

The mouse model of neonatal HI is known to result in extensive cerebral atrophy in retarded neurobehavioral development deficits [20,21]. At 28 d after HI insult, mice showed significant loss of ipsilateral brain tissue (atrophy) and cerebral asymmetry (*P* < 0.01), whereas mice treated with the PB@PDA NPs showed less cerebral atrophy after HI injury compared to vehicle-treated HI mice (*P* < 0.01; Fig. 8A and B).

Following neonatal HI injury, it is crucial to reconstruct neural circuit and maintain neural network homeostasis for neurological recovery. Synaptic protein is required for maintaining synaptic plasticity [22]. To determine whether PB@PDA NPs induce synaptic modifications, we examined the expression of Syn and PSD95, which are hallmark proteins of neuronal synapse, using Western blot analysis and immunofluorescence. Immunofluorescence staining supported also the effect of PB@PDA NP treatment on the expression of PSD95 (Fig. 8C). As shown in Fig. 8D to F, PB@PDA NP treatment increased protein expression of PSD95 and Syn as compared to vehicle-treated HI mice at 28 d after HI (PSD95: *P* < 0.001 and Syn: *P* < 0.05). These results suggested that PB@PDA NP treatment alleviated synaptic damage following HI insult.

### PB@PDA NP treatment improved neurobehavioral impairment following HI insult in neonatal mice

**Fig. 9.**
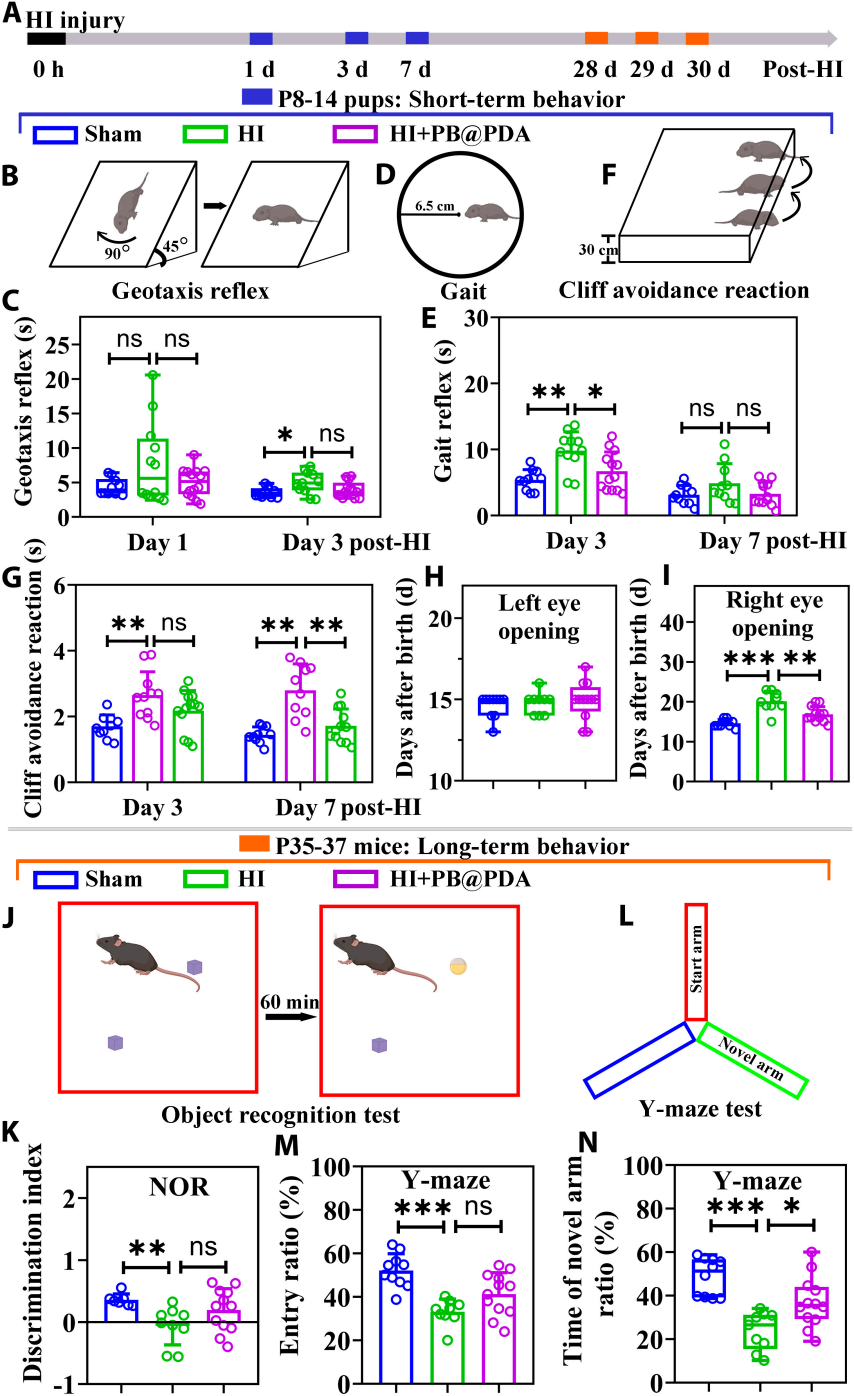
PB@PDA NPs improved short-term and long-term neurobehavioral outcomes following HI injury. (A) Intracardiac administration of PB@PDA NPs improved short-term neurological outcomes 1, 3, and 7 d after HI. HI-caused neurological deficits were evaluated by negative geotaxis reflex (B and C), gait reflex (D and E), cliff avoidance reaction (F and G), and eye-opening time (H and I), compared with the Sham group. A dose of 20 mg/kg of PB@PDA NPs significantly improved neurological function after HI [data are presented as means ± SD of 10 to 14 per group, Kruskal–Wallis ANOVA test in (C), one-way ANOVA followed by post hoc Bonferroni in (E) and (G) (3 d) and (I), and one-way ANOVA followed by post hoc Tamhane T2 in (G) (7 d), *P < 0.05, **P < 0.01, ***P < 0.001]. Intracardiac administration of PB@PDA NPs improved long-term neurological outcomes 28 d after HI. HI-caused neurological deficits were evaluated by NOR test (J and K) and Y-maze (L to N), compared with the Sham group. A dose of 20 mg/kg of PB@PDA NPs significantly improved neurological function after HI [data are presented as means ± SD of 7 to 12 per group, one-way ANOVA followed by post hoc Tamhane T2 in (K) and one-way ANOVA followed by post hoc Bonferroni in (M), and Kruskal–Wallis ANOVA test in (N), *P < 0.05, **P < 0.01, ***P < 0.001].

Perinatal ischemia and/or hypoxia are major risk factors for neurologic injury that often manifest as sensorimotor and locomotor deficits throughout development and into maturity [21]. Furthermore, evidence had indicated that mice suffered various short- and long-term neurological and neurobehavioral deficits after exposure to HI injury [23]. To assess the effect of PB@PDA NP functional recovery following HI brain injury, we evaluated short-term (eyes opening, geotaxis reflex, gait reflex, and cliff avoidance reaction) and long-term [Y-maze performance and new object recognition (NOR)] neurobehavioral outcomes in Sham, HI-vehicle, and HI + PB@PDA groups (Fig. 9A).

The geotaxis reflex was carried out at P8 and P10 (1 and 3 d following HI) to examine short-term outcomes. HI-vehicle mice displayed a significantly longer latency at P10 compared to the Sham group (*P* < 0.05); however, performance was improved in HI + PB@PDA mice at P10 after injury (Fig. 9B and C).

The gait reflex and cliff avoidance reaction were carried out at P10 and P14 (3 and 7 d following HI) to examine short-term outcomes. Performance was impaired at P10 and P14 in HI-vehicle mice for the gait reflex, and PB@PDA NP treatment protected mice from this deficit, significantly decreasing longer latency at P10 (*P* < 0.05; Fig. 9D and E). For the cliff avoidance reaction, we observed that HI-vehicle mice exhibited impaired performance at P10 (*P* < 0.01) and P14 (*P* < 0.001) compared to the Sham group, which was reversed by PB@PDA NP treatment at P14 (*P* < 0.01; Fig. 9F and G). Pups were examined for eye opening every day until P23 and then every 2 h until eye opening. The mean time to the right eye opening was significantly shorter in the PB@PDA NP-treated HI group than in the vehicle-treated HI group (16.8 and 20.1 d for PB@PDA NP-treated HI and vehicle-treated HI, respectively), while that to the left eye opening was not different between each group (Fig. 9H and I).

Previous studies have confirmed that HI injury also displays long-term learning memory deficits [24,25]. Meanwhile, the down-regulation of the expression of synaptic proteins such as Syn and PSD95 also promoted memory functional deficit [22,26]. The NOR and Y-maze was performed at 28 d after injury to measure working memory of mice. Mice in the HI group showed object location memory dysfunction (*P* < 0.05), and PB@PDA NPs did not affect the time duration of exploring the novel object (Fig. 9J and K). Following HI, there were significant reductions in the amount of time (*P* < 0.001) and the ratio entry (*P* < 0.001) in the novel arm of the maze as compared with those obtained in the Sham group. However, PB@PDA NP administration significantly increased both the time (*P* < 0.05) in the novel arm as compared with those of the HI group (Fig. 9L and N and Fig. S16). PB@PDA NP treatment improved neurobehavioral impairment following HI insult in neonatal mice. Taken together, our results demonstrate that PB@PDA NPs have a short-term and long-term effect on brain damage.

### PB@PDA NPs facilitated motor function recovery after stroke in adult mice

**Fig. 10.**
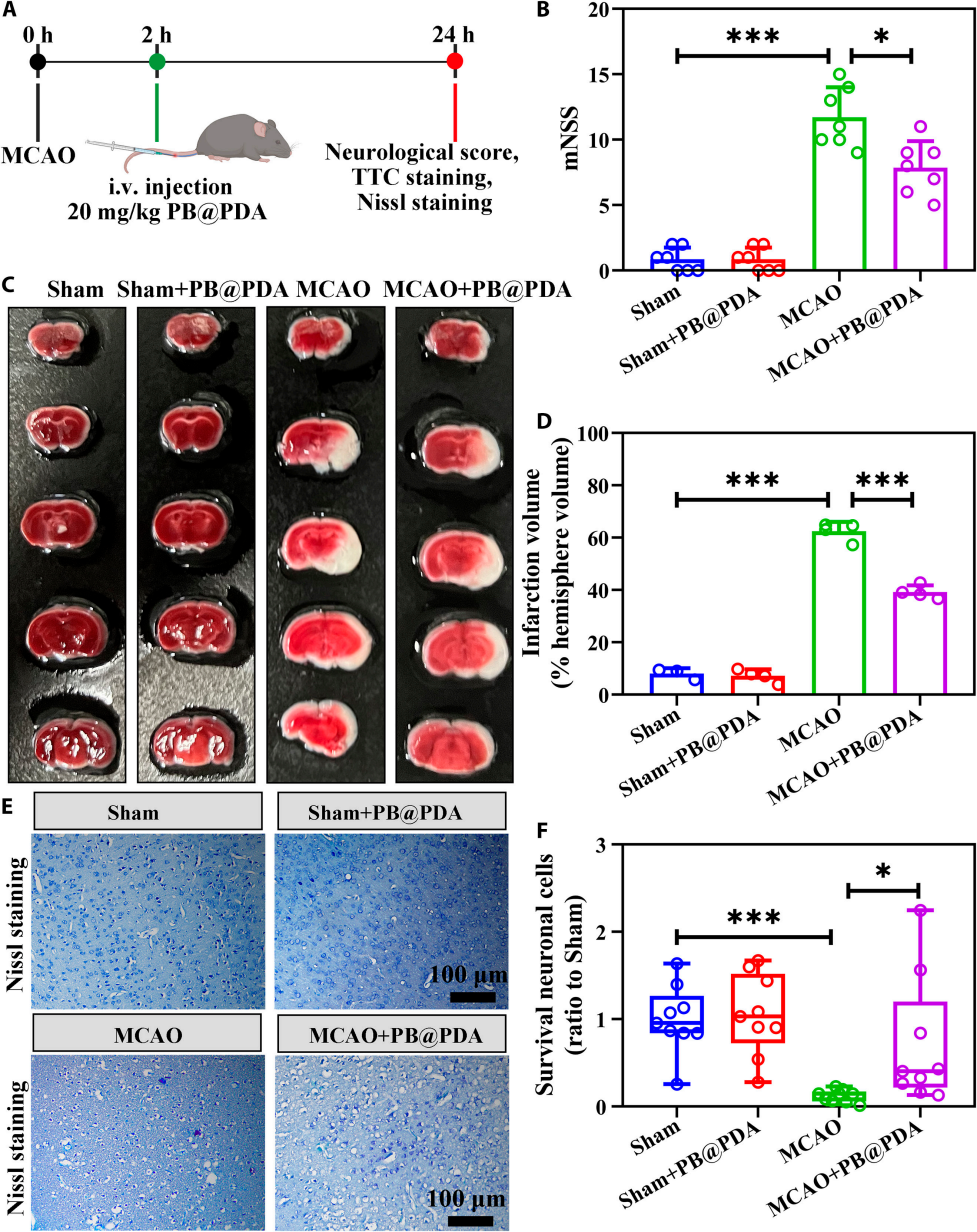
Effect of PB@PDA NPs on anti-ischemic stroke efficacy. (A) Time axis of operation of the animal experiment in the MCAO model. (B) Neurological scores of different treatment groups after MCAO (data are presented as means ± SD of 7 per group, one-way ANOVA followed by post hoc Tamhane T2, *P < 0.05, ***P < 0.001). (C) Images of TTC staining with different treatments. (D) Quantitative analysis of the ratio of the infarct volume to the hemisphere brain volume (data are presented as means ± SD of 4 per group, one-way ANOVA followed by post hoc Bonferroni, ***P < 0.001). (E) Nissl staining of ischemic penumbra sections. Scale bar, 100 μm. (F) Nissl bodies were counted and analyzed in the cortex (data are presented as means ± SD of 9 per group, Kruskal–Wallis ANOVA test, *P < 0.05, ***P < 0.001).

There are similar pathological processes such as oxidative stress, cell apoptosis, and neuroinflammation in neonatal and adult ischemic stroke models [27–29]. To further explore the efficacy of PB@PDA NPs in adult ischemic stroke models, we established a middle cerebral artery occlusion (MCAO) stroke model (Fig. 10A). Behavioral performance was measured at 24 h following ischemia. The Neurological Severity Scores (mNSS) was further investigated to evaluate the neurological functions of mice. A higher score indicated severe damage. Severe behavioral deficits could be observed in the MCAO group (Fig. 10B). The PB@PDA NP group demonstrated the lower score (*P* < 0.05, compared with the MCAO group), indicating optimal protection efficacy. The infarct area with 2,3,5-triphenyltetrazolium chloride (TTC) staining (Fig. 10C and D) reached 60% of the total brain tissue after MCAO. The PB@PDA NP treatment group displayed the lower infarct area of 40% (*P* < 0.001, compared with the MCAO group). Consistent with these findings, analysis by Nissl and H&E assay revealed a decrease in apoptotic cell numbers in PB@PDA NP treatment groups (*P* < 0.05, compared with the MCAO group; Fig. 10E and F and Fig. S17). The above results indicated the PB@PDA NP treatment could efficiently alleviate the injury of the stroke brain and achieve anti-ischemic stroke efficacy in adult period. Age-related differences after stroke have been reported with respect to higher mortality and organ failure, and poor cognition in neonatal HI injury compared to adult stoke [30]. The different mechanism of PB@PDA NPs in neonatal and adult stroke would be further investigated in the future.

## Discussion

At present, therapeutic hypothermia during the incubation period or earlier is widely used in the clinical treatment of neonatal ischemic stroke due to its safety and effectiveness. The main treatment for adult ischemic stroke is intravenous thrombolysis within 4.5 h of onset or interventional thrombolysis within 6 h to restore blood supply in a timely manner clinically [2]. However, the strict and short treatment time window means that only a small proportion of ischemic stroke patients receive timely revascularization therapy. In addition, if thrombolysis and mechanical thrombectomy were performed outside the window of treatment time, the risk of destruction of the blood–brain barrier (BBB) and cerebral hemorrhage will be increased, resulting in increased disability and mortality rate of ischemic stroke. In addition, a series of biochemical reactions induced in the process of restoring blood perfusion can cause ischemia–reperfusion injury, aggravate neuronal necrosis and apoptosis, and lead to impaired nerve function. The small-molecule antioxidant edaravone is still the main neuroprotective agent used in the clinic. However, the short half-life and low bioavailability of edaravone greatly limit its efficacy and clinical application [31,32]. In contrast, nanomedical drugs show obvious advantages in solving the problems of low catalytic activity, short half-life, and poor biocompatibility of conventional drugs and provide a new way to overcome the treatment bottleneck of ischemic stroke.

Intracardiac injection is widely used in the practice for catheter drainage, drug administration, and puncture [33,34]. It is reported that the temporal vein of postnatal (P)1 and P2 neonatal mice is clearly visible when using a dissecting microscope. However, the injection procedure becomes technically unreliable and difficult to observe for the P3 vein [35]. Meanwhile, we found that intracardiac injections did not cause weight loss and death in mice in our previous study [36]. In our current experiments, the PB@PDA NP treatment was carried out in mice at P7. We showed that intracardiac injection of PB@PDA NPs has a good therapeutic effect after brain injury.

Mitochondrial dysfunction in neurons is the major cause of HI brain damage [37,38]. HI exposure markedly reduces the mitochondrial transmembrane potential and elevates ROS generation, leading to brain injury [39].The changes of mitochondrial membrane potential and ROS levels were measured to examine whether the antioxidant effect of PB@PDA NPs was related to ROS scavenging. Microglia are heavily activated to participate in the inflammatory response after HI brain injury [36,40]. Meanwhile, oxidative stress disorder also induces inflammation [41]. After ischemia, microglial activation is initially triggered by neuronal death by releasing products like damage-associated molecular pattern molecules. Damage-associated molecular pattern molecules, which include high-mobility group box 1, extracellular PRX family proteins, and galectin-3, are the main factors that activate microglia [42]. Although we have found that PB@PDA NPs are less distributed within microglia, their inhibition of microglia activation and inflammatory cytokine release may be achieved by improving neuronal apoptosis. In this study, multifunctional PDA-modified PB NPs with ROS responsiveness and mitochondrial-targeted were established for ischemic stroke. PB@PDA NPs have better biocompatibility and antioxidant capacity and can better penetrate the BBB to target the brain compared with PB NPs. This study demonstrated that PB@PDA NPs could target mitochondria, maintain membrane potential, and consume ROS in the mitochondria after ischemic stroke. All these concerted actions alleviated the oxidative stress and cascaded inflammation to restore mitochondrial function and inhibit mitochondria-mediated apoptosis of neurons. Ex vivo fluorescence imaging illustrated the active targeted ability to the ischemic site of PB@PDA NPs in HI mice. The in vivo therapeutic efficacy also illustrated that the PB@PDA NPs could greatly ameliorate mNSS and decrease infarct volume in response to the surgical MCAO injury in adult mice.

Base on in vivo experiments, we found that PB@PDA NPs can reach the heart, liver, spleen, lung, and kidney, beside the brain. Among these organs, PB@PDA NPs concentrated more in the liver. Thus, whether PB@PDA NPs affect mitochondria of other organs, we selected liver cells as representative to observe the colocalization of PB@PDA NPs with mitochondria of other cells in vitro. The results showed that PB@PDA NPs could also colocate with mitochondria of AML12 cell. Moreover, PB NPs possess antioxidant activity and play an importantly role in preventing against liver injury. For example, manganese PB nanozymes prevent acute liver injury by attenuating oxidative stress and regulating inflammation [43,44]. We found that the PB@PDA NPs had no toxicity in the liver by H&E staining and biochemistry assays. Together, we speculated that PB@PDA NPs can improve stroke-induced liver injury for targeting mitochondrion of liver cells. Future studies are needed to identify PB@PDA NP-mediated mitochondrial functions in each organ and to examine how these mitochondrial functions relate to complications of stroke. This provides a reference for the subsequent application of PB@PDA NPs in other diseases.

Dopamine molecules contain catechol and amino functional groups in the structure of adhesion proteins secreted by mussels. Under alkaline conditions, dopamine molecules can be deposited on the surface of organic and inorganic materials through oxidative self-polymerization to form a PDA coating layer. PDA has strong adhesion properties similar to adhesion proteins. The catechol and amine groups of PDA NPs collectively contribute to the binding of NPs to D2 dopamine receptors (D2DRs), and their uptake by D2DR-expressing cell types in vitro [45]. The results of our experiment found that the fluorescent signals in the mouse brains were stronger and prolonged duration, while negligible fluorescence was found in other organs such as the heart, lung, liver, and kidney at 24 h after intracardiac administration of PB@PDA-ICG NPs. It is widely known that neurons express D2DR [46]. Thus, PB@PDA NPs can colocalize with the neuron, similarly as we observed in the present experiment. In addition, the BBB is destroyed after stroke [47,48], and PB@PDA NPs entered the blood circulation after intracardiac administration and easily accumulated the brain through damaged BBB. Together, PB@PDA NPs accumulate more in the brain instead of adhering peripheral organs.

Thus, PB@PDA NPs, which have good antioxidant activity and biocompatibility, are easy to prepare and modify, and exerted synergistic antioxidant effect to rescue damaged neurons from further damage in neonatal and adult ischemic stroke models. We believe that this nanoplatform represents a promising strategy for the treatment of ischemic stroke. Besides, the fabrication of multifunctional NPs for other brain diseases needs to be investigated in the future.

## Data Availability

The data that support the findings of this study are available from the corresponding author upon reasonable request.

## Supplementary Materials

Supplementary 1 Supplementary Text Figs. S1 to S17 Table S1 References
