# Supplementary material for: Polydopamine-Cloaked Nanoarchitectonics of Prussian Blue Nanoparticles Promote Functional Recovery in Neonatal and Adult Ischemic Stroke Models
Source: Biomater Res. 2024 Sep 18;28:0079. doi: 10.34133/bmr.0079 (PMC11409202; doi:10.34133/bmr.0079)
Supplement: Supplementary 1 — Supplementary Text Figs. S1 to S17 Table S1 References [file bmr.0079.f1.zip › Supporting Information .docx]

Supporting Information

**Polydopamine-Cloaked Nanoarchitectonics of Prussian Blue Nanoparticles Promote Functional Recovery in Neonatal and Adult Ischemic Stroke Models**

Yijing Zhao^1#^, Cong Song^2#^, Haijun Wang^2#^, Chengcheng Gai^1^, Tingting Li^1^, Yahong Cheng^1^, Junjie Liu^2^, Yan Song^1^, Qian Luo^3^, Bing Gu^3^, Weiyang Liu^4^, Liwei Chai^1^, Dexiang Liu^3*^, and Zhen Wang^1**^

**Experimental Section**

***Materials.*** Potassium ferricyanide, polyvinylpyrrolidone (PVP, K30), and 3,3′,5,5′-Tetramethylbenzidine (TMB) were purchased from Shanghai Aladdin Biochemical Technology Co., Ltd. (Shanghai, China). Hydrochloric acid (HCl, aq.) was from Yantai Yuandong Fine Chemical Co., Ltd. (Shandong, China). Tris-HCl and dopamine hydrochloride (DA.HCl) were obtained from Sinopharm chemical Reagent Co., Ltd. (Shanghai, China). Fluorescein isothiocyanate (FTIC) and indocyanine green (ICG) were obtained from Shanghai Hongye Biochemical Technology Co., Ltd. (Shanghai, China). DMEM, DMEM/F12, Neurobasal, fetal bovine serum (FBS), Trypsin-EDTA (0.5% trypsin, 5.3 mM EDTA tetra-sodium), B27, L-Glutamine and antibiotic penicillin and streptomycin (P/S, 100 U/mL) were obtained from Gibco BRL (Ontario, Canada). Phenylmethanesulfonylfluoride (PMSF), Dihydroethidium (DHE), 4’,6-diamidino-2-phenylindole (DAPI), 2’,7’-dichlorifluorescein diacetate probe (DCFH-DA, S0033M) and MitoTracker Red fluorescence probe were purchased from Beyotime (Shanghai, China). Mitochondrial membrane potential assay kit with JC-1, cell counting kit-8 assay and MitoSOX Red Mitochondrial Superoxide Indicator were purchased by Yeasen (Shanghai, China). Isoflurane and suture for middle cerebral artery (MCA) occlusion (MCAO) model were purchased from Shenzhen Ruiward Life Technology Co., Ltd (Shenzhen, China). The 2,3,5-triphenyltetrazolium chloride (TTC) staining were obtained from Sigma (MA, USA). Diaminobenzidine (DAB), Terminal-deoxynucleoitidyl Transferase Mediated Nick End Labeling (TUNEL), Nissl and Hematoxylin and Eosin (H&E) staining were purchased by Servicebio (Wuhan, China). Anti-β-actin (#TA-09), Horseradish Peroxidase-labeled AffiniPure Goat Anti-Rabbit IgG (H+L) and Anti-Mouse IgG (H+L) were obtained from ZSGB-BIO (Beijing, China). Arginase-1 Antibody (#9819), Cleaved Caspase-3 (Asp175) Antibody (#9661) were purchased by Cell Signaling Technology (Boston, MA, USA). Anti-Iba-1 antibody (#019-19741) were purchased by Wako Pure Chemicals (Tokyo, Japan). Anti-NeuN antibody (#ab104224) were purchased by Abcam (Cambridge, MA, USA). Caspase 3/p17/p19 Polyclonal antibody (#19677-1-AP), GFAP Monoclonal antibody (#60190-1-Ig), HO-1/HMOX1 Polyclonal antibody (#10701-1-AP), iNOS Polyclonal antibody (#22226-1-AP), PSD95-Specific, DLG4 Polyclonal antibody (#20665-1-AP), SOD2 Polyclonal antibody (#24127-1-AP), Synaptophysin Monoclonal antibody (#60191-1-Ig) were obtained by Proteintech (Rosemont, IL, USA).

***Study on peroxidase-like activity of NPs.*** First, solutions of TMB (10 mg/mL, DMSO), H_2_O_2_ (30%, water), PB, PDA and PB@PDA NPs (20 μg/mL, PBS) were separately prepared. Then, the solutions with equal amounts were mixed in order according to the following groups: PB+H_2_O_2_+TMB, PDA+H_2_O_2_+TMB, PB@PDA+H_2_O_2_+TMB, TMB. After 15 min, UV-vis spectra were recorded to observe the change in the absorbance at 650 nm associated to TMB.

***Animals*.** All animal care and experimental procedures were conducted in accordance with the guidance of the Care and Use of Laboratory Animals from the National Institutes of Health and were approved by the Laboratory Animal Ethics Committee of Shandong University (approval No. ECSBMSSDU2022-2-52). C57BL/6J mice were purchased from the Beijing Vital River Laboratory Animal Technology (license No. SCXK (Jing) 2021-0006). All animals were maintained in individual cages of standard Specific Pathogen Free conditions and maintained in individual cages of constant 20-25 °C temperature and 45-55% humidity under a 12:12 h light/dark cycle. In this study, each litter was specified to contain one female and 8–10 pups. For hypoxia-ischemia (HI) model, each litter was specified to contain one female and 8-10 pups postnatal (P)7 days in this study. Gait analysis was used to assess motor function in P7 pups, and only male pups with normal motor function were used in the experiment. For MCAO model, male mice weighing 22-24g with a modified neurological severity score of 0 were used in the experiment.

***Hemolysis Assay.*** The hemocompatibility of PB@PDA NPs according to the literature^[25]^. In brief, fresh mouse blood was placed in a centrifuge tube for centrifugation (3000 rpm/min, 10 min), and then the supernatant was discarded and the precipitate was diluted with PBS. Repeat the operation for more than 3 times to obtain pure red blood cells (RBCs). The obtained RBCs were resuspended with 5 mL of PBS. Afterwards, 100 μL of the obtained RBC suspension was mixed with 900 μL PBS (negative control), 1% TX-100 (positive control) or PBS solutions of PB@PDA NPs with different concentrations (1, 2, or 3 mg/mL), respectively. After 2 h incubation at 37℃, all the samples were centrifuged at 10000 rpm for 5 min. Finally, the photos of the samples were taken and the absorbance of the obtained supernatant at 540 nm was measured via UV-vis spectrometry. The hemolysis rate was calculated according to the following formula: hemolysis rate (%) = [(A_1_－A^－^) / (A^+^－A^－^)] × 100%. Among them, A_1_ represents absorbance value of sample after hemolysis in 540 nm, A^－^ and A^+^ represent absorbance value of negative control and positive control after hemolysis in 540 nm.

***FTIC or ICG labeled PB@PDA NPs*.** Certain amounts of fluorescent dyes (FITC or ICG) were added to PB@PDA NPs solution. After magnetic stirring for 12 h in dark environment, the mixed solution was centrifugated (12000 g, 2 min) and washed with water for 5 times until the supernatant is free of fluorescent dye. It was detected by small animal in vivo fluorescence imager.

***HI model*.** The most used experimental model of HI was described by Vannucci and coco-workers and modified by Rice^[1]^. The HI model was established as previous research methods^[2]^. The P7 pups were first anesthetized with 2% isoflurane and fixed in supine position. Next, the right common carotid artery was exposed and ligated with 4-0 surgical silk (pre-moistened in PBS solution). After 30 min of recovery, the pups were placed in anoxic chamber (8% oxygen and 92% nitrogen) for 1 h at 37℃, and then put back to their cages. For the sham group, other pups from the same cage were anesthetized and their carotid arteries exposed, but not ligated.

***MCAO model*.** The MCAO model was established as previous research methods^[3]^. Firstly, anesthetize the animal with 2% isoflurane-anesthesia. Briefly, mice were placed supine, a midline incision made in the neck, and the right common carotid artery (CCA), internal carotid artery (ICA) and external carotid artery (ECA) were surgically exposed under a microscope. Insert a filament (usually made of nylon or coated with silicone) into the external carotid artery and advance it to the origin of the MCA to block blood flow. After 2 h, the suture was withdrawn to allow MCA reperfusion. Sham mice underwent the same procedures except for the occlusion of the MCA. Lastly, the incision was closed with sutures, and the animals were then placed into a warm container at 37°C to recover.

***Drug administration.*** The PB, PDA and PB@PDA NPs diluted by Dulbecco’s modified eagle medium/F12 (DMEM/F12). For the treatment groups, the PB, PDA and PB@PDA NPs respectively were administered by intracardiac injection for 2 h after HI. And the PB@PDA NPs was administered through tail vein injection at 2 h after MCAO. The same volume of DMEM/F12 was received by the Sham and HI groups as the treatment groups.

*Long-term behavioral tests.* Behavioral tests were performed 28 days after the HI model (P35). The sequence of behavioral experiments in this study was novel object recognition and Y-maze test. All behavioral experiments were conducted in standard behavioral testing rooms. The behavioral tests were completed by two experimenters who did not know the specific experimental groups. The mice adapted to the behavioral rooms 3 days before the behavioral experiments. And before each behavioral session, the maze was sprayed with 75% ethanol to eliminate residual odors. The Y-maze has three identical arms at an angle of 120°. It consists of a 5 min training session and a 5 min testing session. In the first session, one of the arms (novel arm) is blocked by a blockage, leaving only the other two arms open (start arm and known arm). The mice were placed from the start arm and let them explore the maze undisturbed for 5 min. After 30 min, in the second session, each mouse was replaced from the start arm with the novel arm open. The ratio of time in the novel arm and the number of times entering the novel arm were recorded using a camera and analyzed with SMART 2.5 software (SMART 2.5, Panlab, USA). The novel object recognition test was carried out in a wooden box (40×40×40 cm) with a bottom divided equally into 16 squares consists of three stages. Use two identical objects as the familiar object and the sphere as the new object. Firstly, the mice were placed from the central area of the box and allowed to explore unrestricted for 5 min. Secondly, two identical objects were placed at the bottom of the box, then the mice were placed again and allowed to continue exploring for 10 min. Lastly, one of the objects was replaced with a new one, and the mice were placed in the box for another 10 min. The Smart software was used to record the time of the mice at each stage to recognize different objects.

***Short-term behavioral tests.*** Short-term neurobehavioral deficits were assessed by a blinded investigator at 1 day, 3 days and 7 days after HI, using geotaxis reflex, gait and cliff avoidance reaction, as previously described^[4]^. Geotaxis reflex: The animals were placed on a board tilted 45° with their heads facing down, and the time the animals rotated 90° was recorded, repeated three times. Gait: the animals were placed in the center of a white paper circle of 13 cm in diameter, and the day they began to move off the circle with both forelimbs was recorded. In cases the animal did not leave the circle for 30 s, the test was negative. Cliff avoidance reaction: place the animals on a 30 cm high board in the center, exposing its head and forelimbs to the outside, and record the time it takes the animals to put both forelimbs back on the board. If more than 30 seconds, this test is invalid.

***Modified neurological severity score*.** A neurological examination was performed by a blinded investigator at 24 h after MCAO as previously described^[5]^. The scoring system consists of 6 categories (spontaneous activity, symmetry in the movement of four limbs, forepaw outstretching, climbing, body proprioception, response to vibrissae touch). with each category assigned a score ranging from 0 to 3. All scores were added to get a final score from 0 (normal) to 18 (most severe deficits).

***TTC staining and infarct volume calculation*.** Infarction was determined by TTC staining 72 h after HI and 24 h after MCAO. The brain was immediately removed on ice and placed at -20℃ for 20 min. Then, the brain was quickly cut into four 2-mm-thick coronal slices, which were completely immersed with 2% TTC solution and incubated at 37°C for 15-30 min. Next, the slices were removed in order, and recorded with a camera. Images of these slices were analyzed to obtain infarct area by ImageJ 1.52a. Infarct volume content was assessed using the following formula: Infarct volume (%) = ｛[$(\sum S1+S2+S3+S4$)-$(\sum I1+I2+I3+I4)$]/$(\sum S1+S2+S3+S4$)｝× 100%. *I* 1-4 represents respectively the area of each slice of the ipsilateral non-infarcted tissue, and *S* 1-4 represents respectively the contralateral whole hemisphere area of each slice.

***Brain atrophy measurement.*** At 28 days after HI, the brains of the mice were removed intact and placed on ice. The left and right hemispheres of the brain were weighed separately and recorded as WL and WR respectively. Brain atrophy (%) = (WL-WR)/(WL+WR) *100%.

***Reactive oxygen species (ROS) and MitoSox staining.*** Briefly, the Dihydroethidium and MitoSox solution were diluted in DMSO to a final concentration of 1 mmol/L, and the solution was stored at -20℃ in a dark condition. The frozen section after fixation with 4% paraformaldehyde (PFA) was then stained with DHE and MitoSox solution in the dark. After 37°C incubation for 30 min, it followed that superfluous probe was removed with three-times PBS washing. Fluorescent images were captured when the excitation wavelength was set to 594 nm.

***Nissl and H&E staining*.** The mouse whole brain was soaked in 4% PFA for 24 h, then embedded in paraffin, and sectioned coronally into 4-μm-thick sections for staining. Next, the procedure was carried out according to the instructions of Nissl and H&E staining kits. Stain with Nissl stain solution and H&E stain at room temperature. Finally, the section is scanned using a microscope. The number of Nissl bodies on each slice was recorded using ImageJ 1.52a software.

***TUNEL and Immunofluorescence co-staining*.** The mouse whole brain tissue in 4% PFA solution and routinely dehydrate and embed. The section was washed with PBS and permeabilized with 1% Triton X-100, and then incubated with 10% normal goat serum. Sections were then incubated with TdT enzyme reaction solution for 1-2 h at 37°C. The sections were stained with anti-NeuN (1:500), anti-Iba-1 (1:500) or anti-GFAP (1:500) antibody overnight after soaking in PBS. Then, secondary antibodies (Alexa Fluor 488 or 594, 1:200) were incubated with sections for 1 h protected from light. After counterstaining with DAPI, the tablets sections sealed with anti-fluorescence quencher sealing solution. Photos were taken under a fluorescence microscope (Olympus vs120, Tokyo, Japan).

***Immunohistochemical analysis*.** In the study, we used an immunohistochemical assay to observe microglia in the cortex. After 10-μm-thick paraffin sections were dewaxed and dehydrated, nonspecific binding was blocked by peroxidase block. After washing 3 times with PBS, the sections were incubated overnight at 4℃ with anti-Iba-1 primary antibody diluted by PBS at 1:500. On the second day, DAB staining was performed after incubating the secondary antibody. The color changes of the tissues were observed while incubating. Finally, the sections were sealed with glycerol jelly mounting medium. Microglia take up the staining and express themselves in brown shades. The microglia score was between 0 (no activation) and 4 (total phagocytic activation).

***ROS and Mitochondrial membrane potential measurement.*** The brain ROS and mitochondrial membrane potential were evaluated the level of ROS after treatment with PB@PDA NPs by DCFH-DA fluorescent probe staining, MitoSox-Red fluorescent probe and JC-1 staining. The fluorescence intensity of JC-1 monomer/complex was measured at excitation/emission: 490/530 nm and 525/590 nm. The fluorescence intensity of DCFH-DA was measured at excitation/emission: 488/525 nm. The fluorescence intensity of MitoSox-Red was measured at excitation/emission: 510/580 nm.

***Aspartate transaminase (AST), alanine aminotransferase (ALT), and blood urea nitrogen (BUN) measurement*.** Serum was collected after the mice were sacrificed post HI 28 days. And serum was commissioned to Sevierbio (Wuhan, China) for ALT, AST, and BUN testing (Order number: 856390831851630592).

***Primary cultured neuron, microglia, and astrocytes*.** The P1 pups were sacrificed under deep anesthesia with 2% isoflurane and sterilized with 75% alcohol and craniotomy was performed under sterile conditions. The brain tissue was removed and washed twice with pre-cooled PBS. The meninges and blood vessels in the brain tissue were carefully dissected, and the rest of the brain tissue was cut into pieces and placed into a petri dish with DMEM/F12 high glucose medium. And the same amount of 0.25% trypsin was added and digested at 37℃ for 15 min. Then the cell suspension was filtered through a 70 μm cell filter after the serum stops digesting. After collecting the filtrate, the cell precipitate was obtained by centrifugation at 1500 × g for 10 min. Then, the cells were resuspended, about 1 × 10^5^ cells were plated onto each well of 24-well plates for TUNEL staining, and 1 × 10^4^ cells were plated onto each of 96-well plates for Cell counting kit-8 (CCK8) assay. The neurons were then put into a humidified incubator with 5% CO_2_ at 37 °C. The medium was changed to neurobasal medium supplemented with 2% B27 (maintenance medium) after 6 h. For primary microglia, briefly, coat two T-25 culture flasks with 2 mL each of 10 μg/mL poly-L-lysine for 2 h. And the cell precipitation was resuspended and inoculated into culture bottles with DMEM/F12 high-glucose complete medium. After 24 h, all the mediums were changed, and then the solution was changed once 3 days later, until the glial cells were stratified at 7-9 days. The upper cells are round or oval, mainly microglia and oligodendrocytes, and the lower cells are mainly neurons and astrocytes. Finally, after cell stratification, the culture flask was sealed and placed on a constant temperature shaker at 37℃ for 2 and 24 h (250 r/min), and the upper cells were harvested as primary microglia by centrifugation and continued in cell incubator.

***CCK8 cell viability assay*.** The primary neuron, microglia and astrocytes were treated with different concentrations of PB@PDA NPs for CCK8 experiment. After treatment, add CCK8 solution to each well. CCK8 reagent is highly sensitive, so a typical ratio is 10 μL of CCK-8 solution to 100 μL of culture medium. Gently shake the plate to ensure thorough mixing of the CCK-8 reagent with the culture medium. Return the plate to the incubator and continue to incubate, typically for 2 h. Measure the absorbance (OD value) inside the wells using a microplate reader, commonly at a wavelength of 450 nm.

***MitoTracker staining*.** Primary neuron or AML12 cell was seeded in 24-well plates at a density of 1 × 10^5^ cells per well. Subsequently, 500 μL of MitoTracker staining working solution was thoroughly mixed with the cells. The cells were then incubated at 37°C for 20 min in a cell culture incubator, and stained with DAPI solution for 5 min. The immunofluorescence image directly observed by a fluorescence microscope.

***Western blot Analysis*.** Total protein from pups’ brain cortex tissues 3 days after HI was acquired using mixture of RIPA Lysis Buffer, PMSF Solution and Phosphatase inhibitor (mixing ratio: 100:1:1). The concentration of protein was determined using the diquinolinic acid protein assay kit. Equal amounts of protein (20 μg) contained with sodium dodecyl sulfate polyacrylamide gel electrophoresis sample loading buffer from brain tissue were separated, and then transferred to 0.45 μm PVDF transfer membranes (Millipore, USA). The membranes were blocked with 10% skim milk for 1 h and incubated with primary antibodies at 4°C overnight, including HO-1 (1:1000), SOD2 (1:1000), iNOS (1:1000), Arg-1(1:2000), Cleaved Caspase 3 (1:1000), Caspase 3 (1:1000), PSD95 (1:1000), Syn (1:5000), and β-actin (1:3000). On the second day, anti-rabbit or anti-mouse secondary antibodies were incubated at room temperature for 1 h. Finally, the membranes were covered with ECL luminol reagent and imaged using Chemiluminescence imaging system (Tanon-5200, Shanghai, China).

**Supplement Figures and Tables**

**
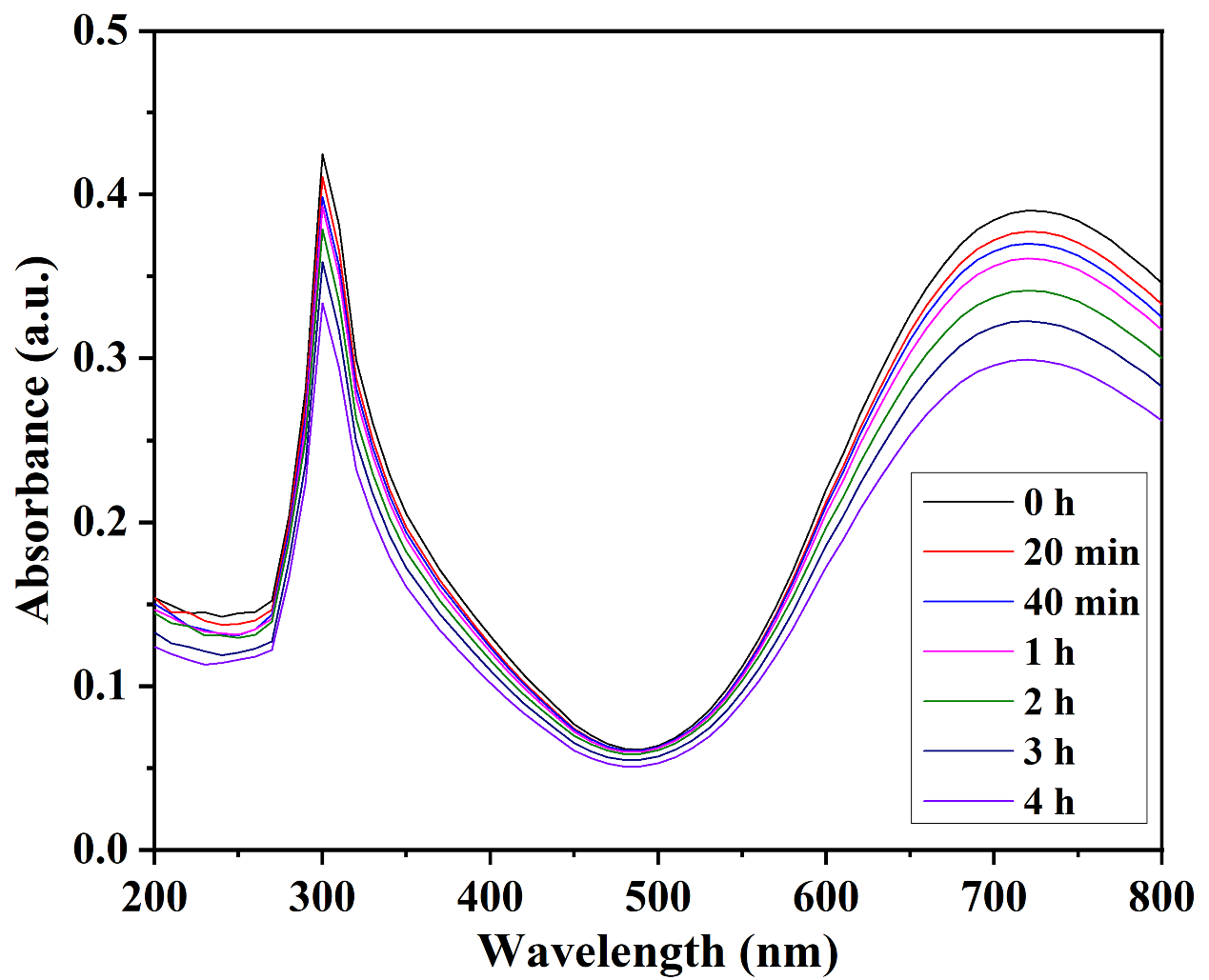
**

**Figure S1.** UV-Vis absorption spectra of PB NPs in Tris-HCl buffer solution (pH=8.5, 10 mM) for 20 min, 40 min, 1 h, 2 h, 3 h and 4 h.

**
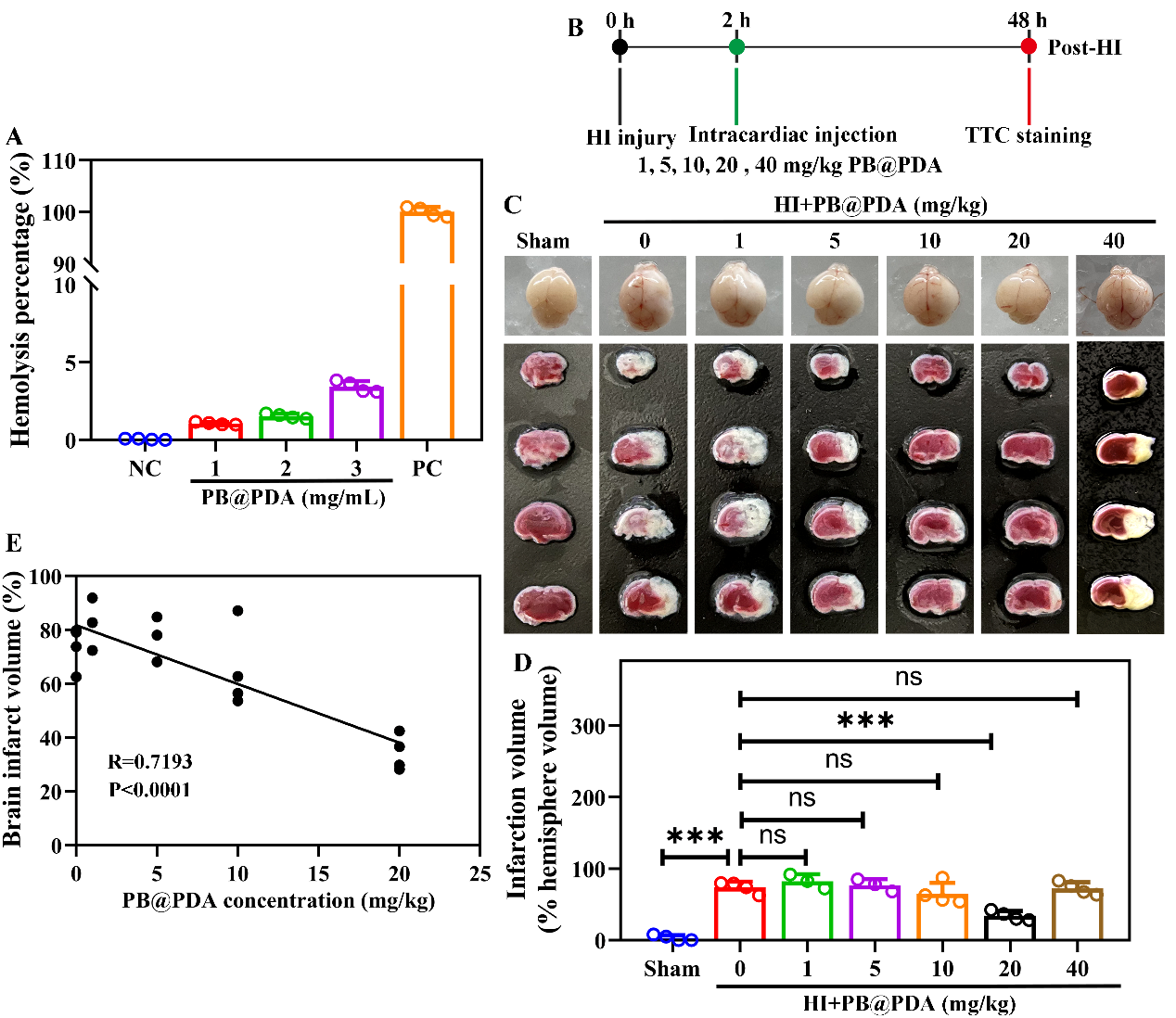
**

**Figure S2.** (A) Hemolysis percentage of the mouse treated with PB@PDA NPs under the concentration of 1, 2, and 3 μg/mL, respectively. 1% TX-100 as a positive control, and PBS as a negative control. (B) Treatment schedule. Different concentrations of PB@PDA NPs (1mg/kg, 5 mg/kg, 10 mg/kg 20 mg/kg or 40 mg/kg) was intravenously injected intracardially into mice on 2 h after HI. TTC staining was conducted on 48 h post-HI. (C) Representative samples stained with TTC at 48 h following HI insult under different concentrations of PB@PDA NPs treatment. (D) Infarct volumes were calculated as the percentage of infarct volume to hemisphere brain (Data are presented as means ± SD of 3-4 per group, one-way ANOVA by post-hoc Bonferroni, ns: not significant, ****p* < 0.001) (E) Positive correlation (R=0.7193, P<0.0001) between brain infarct volume and PB@PDA concentrations


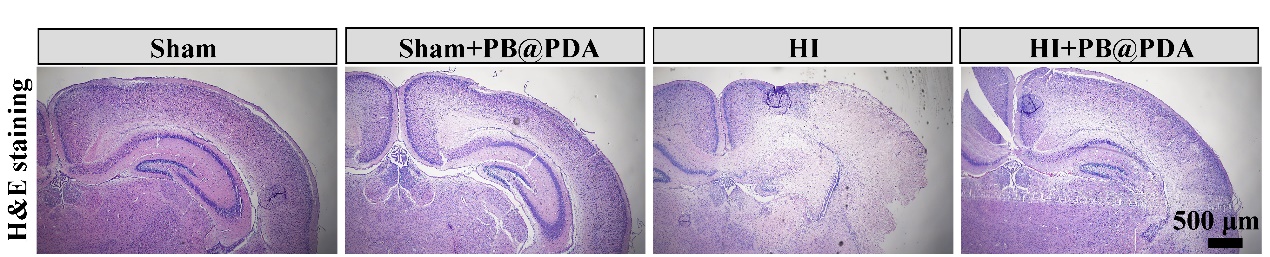


**Figure S3.** H&E staining of brain tissues at 48 h post-HI. Scale bar: 500 μm.

**
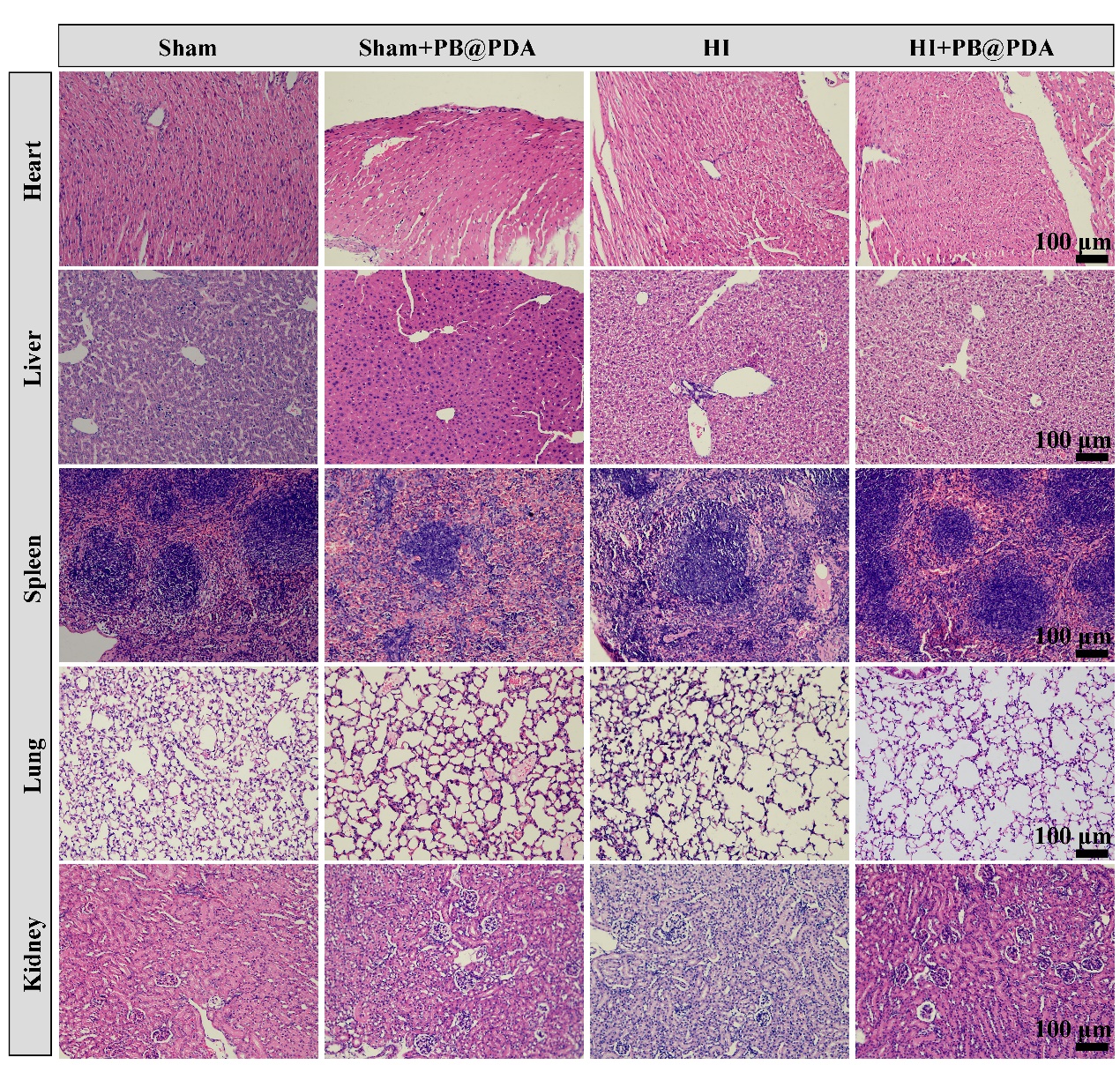
**

**Figure S4.** H&E staining of tissues from the major organs of mice treated with and without PB@PDA at 28 days post-HI. Scale bar: 100 μm.

**
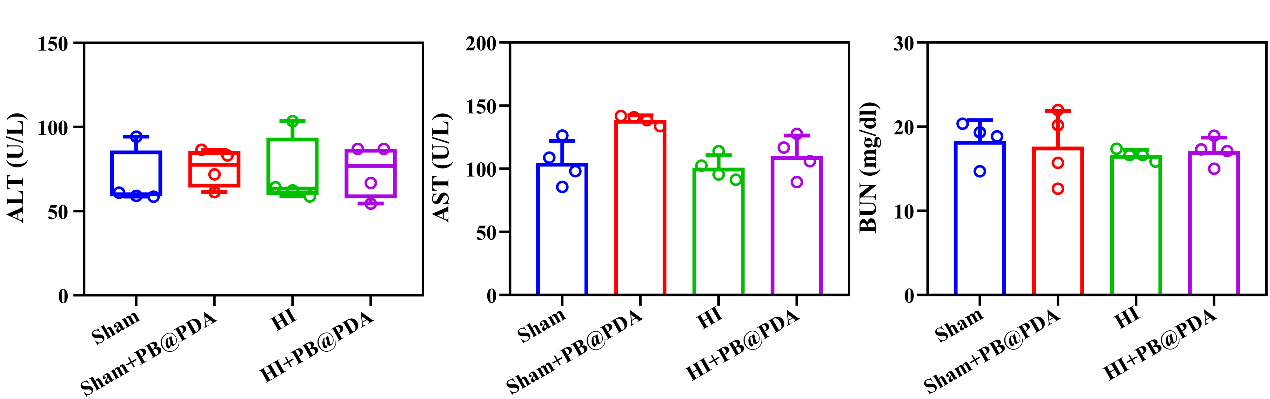
**

**Figure S5.** ALT, AST, and BUN were measured via ELISA kits in the serums of the mice 28 days post-HI (Data are presented as means ± SD of 3 per group).

**
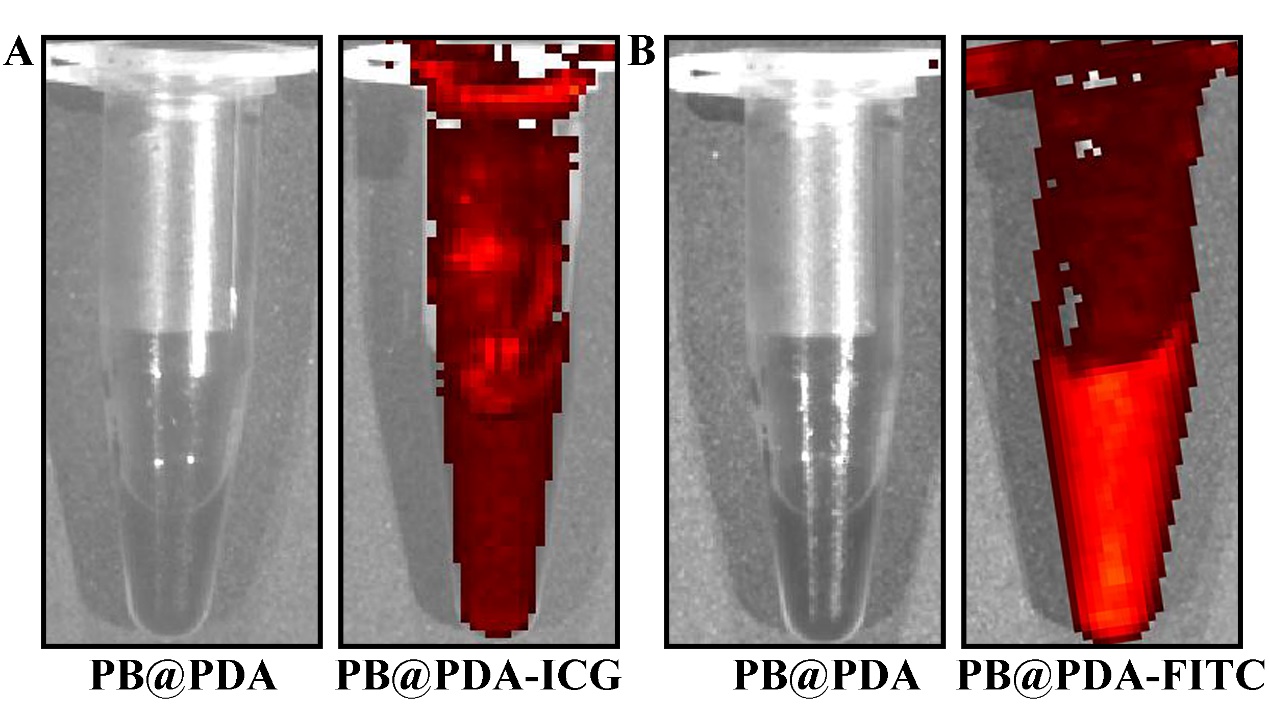
**

**Figure S6.** (A) The fluorescence images of PB@PDA NPs, and PB@PDA -ICG NPs. (B) The fluorescence images of PB@PDA NPs, and PB@PDA -FITC NPs.

**
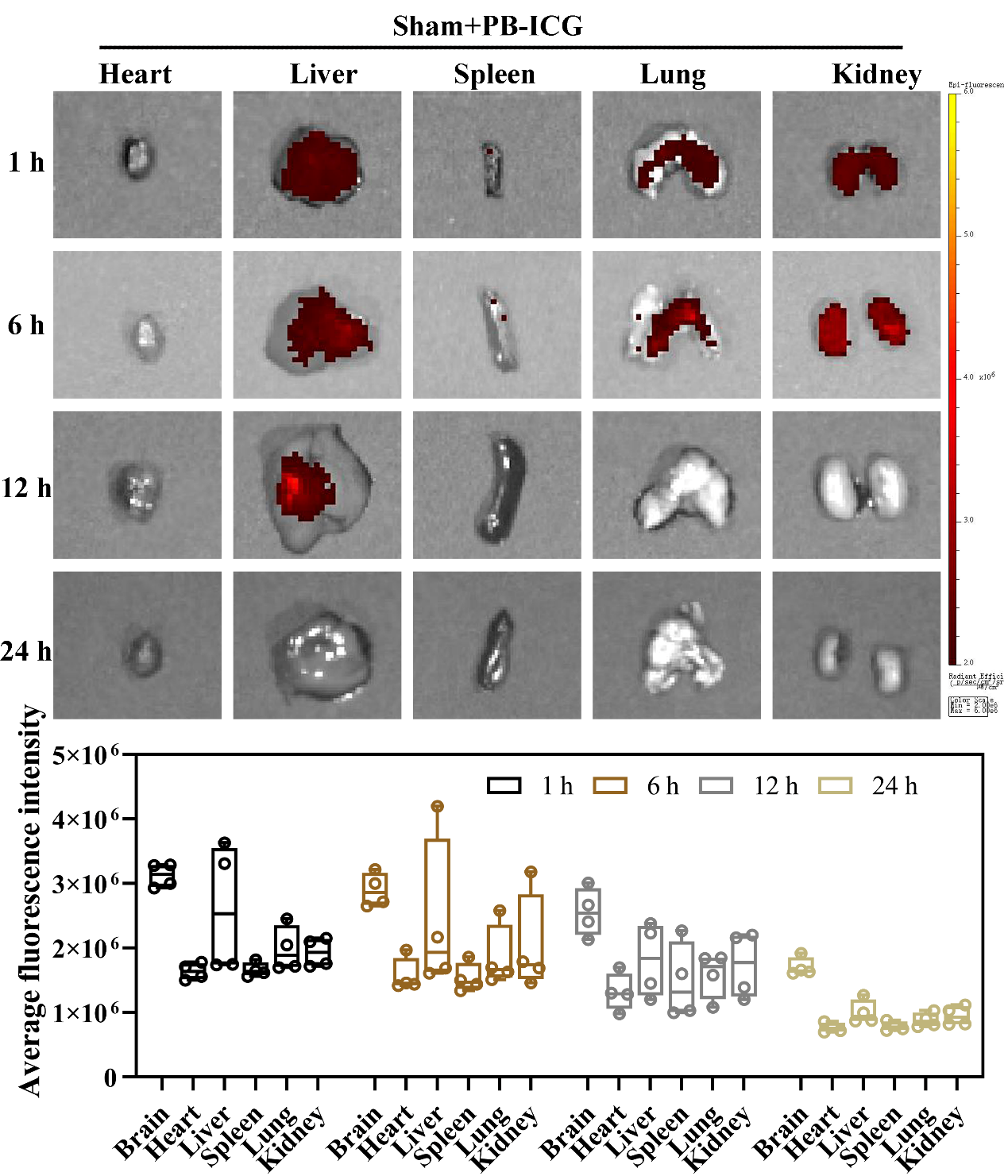
**

**Figure S7.** (A) Fluorescence imaging of brain and main organs from mice in the Sham+PB group at 1 h, 6 h, 12 h, and 24 h after intracardiac injection with ICG-labeled PB NPs. (B) The fluorescence level of the main organs was quantified by the mean fluorescence intensity (Data are presented as means ± SD of 4 per group).

**
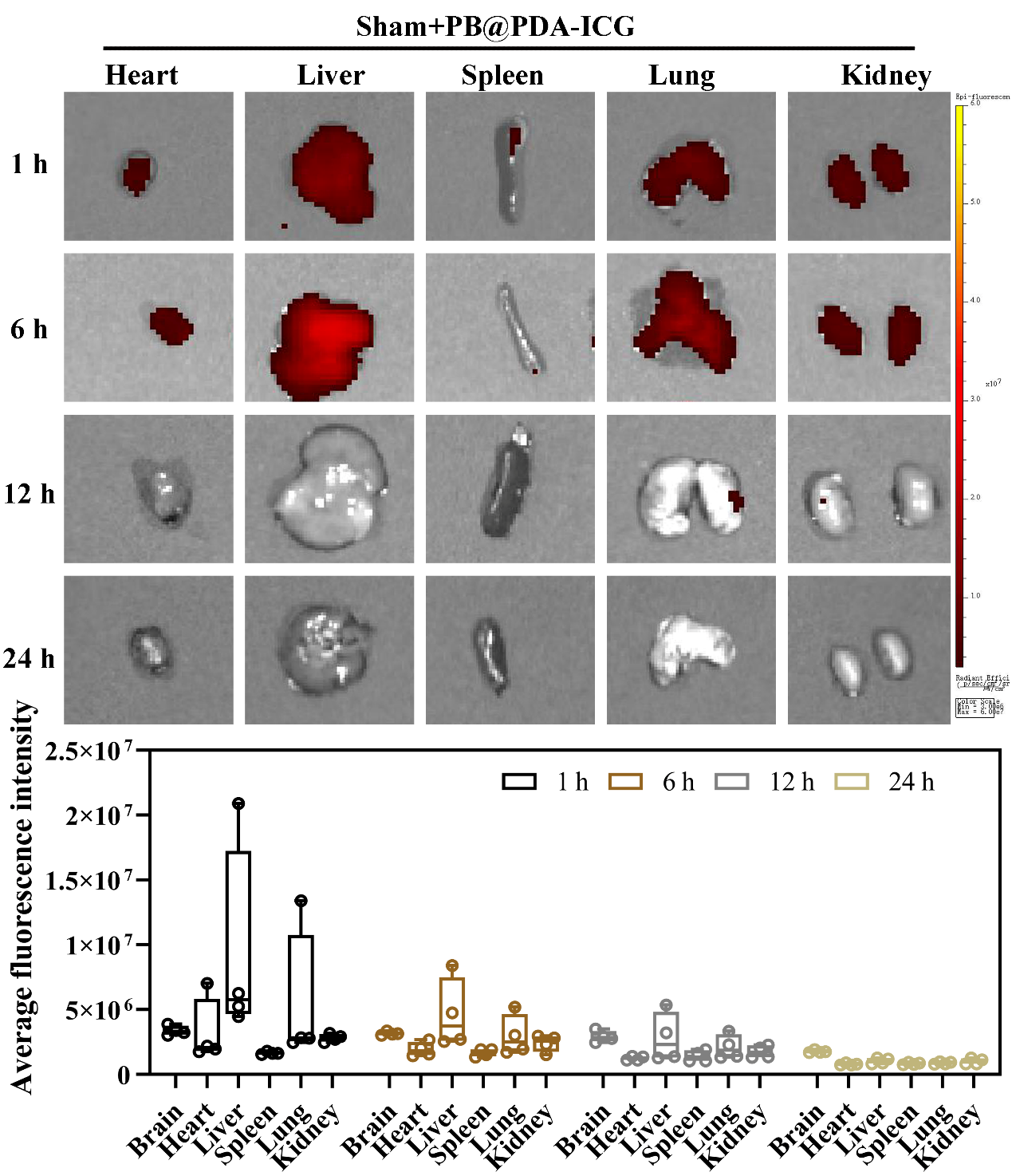
**

**Figure S8.** (A) Fluorescence imaging of brain and main organs from mice in the Sham+PB@PDA group at 1 h, 6 h, 12 h, and 24 h after intracardiac injection with ICG-labeled PB@PDA NPs. (B) The fluorescence level of the main organs was quantified by the mean fluorescence intensity (Data are presented as means ± SD of 4 per group).

**
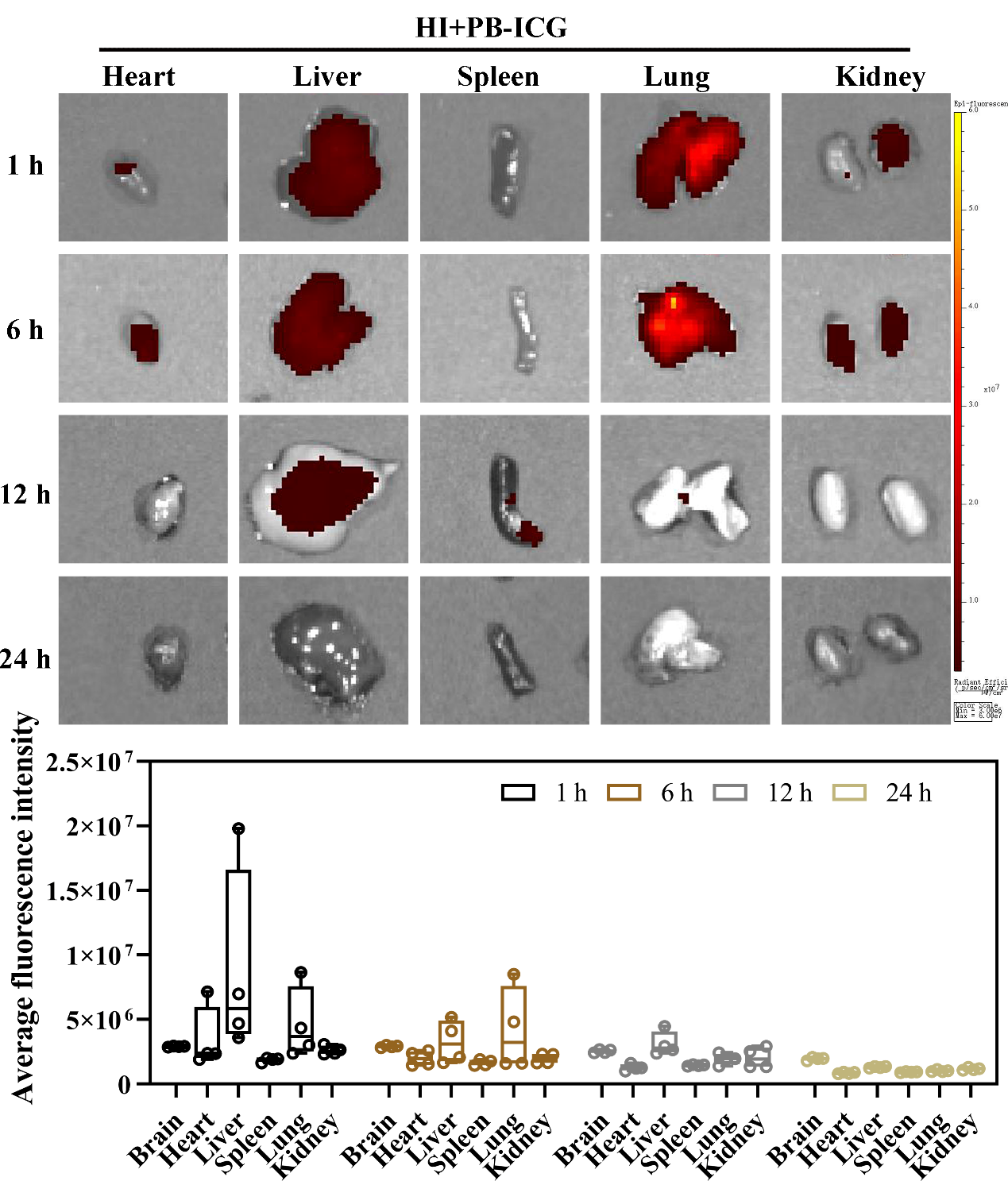
**

**Figure S9.** (A) Fluorescence imaging of brain and main organs from mice in the HI+PB group at 1 h, 6 h, 12 h, and 24 h after intracardiac injection with ICG-labeled PB NPs. (B) The fluorescence level of the main organs was quantified by the mean fluorescence intensity (Data are presented as means ± SD of 4 per group).

**
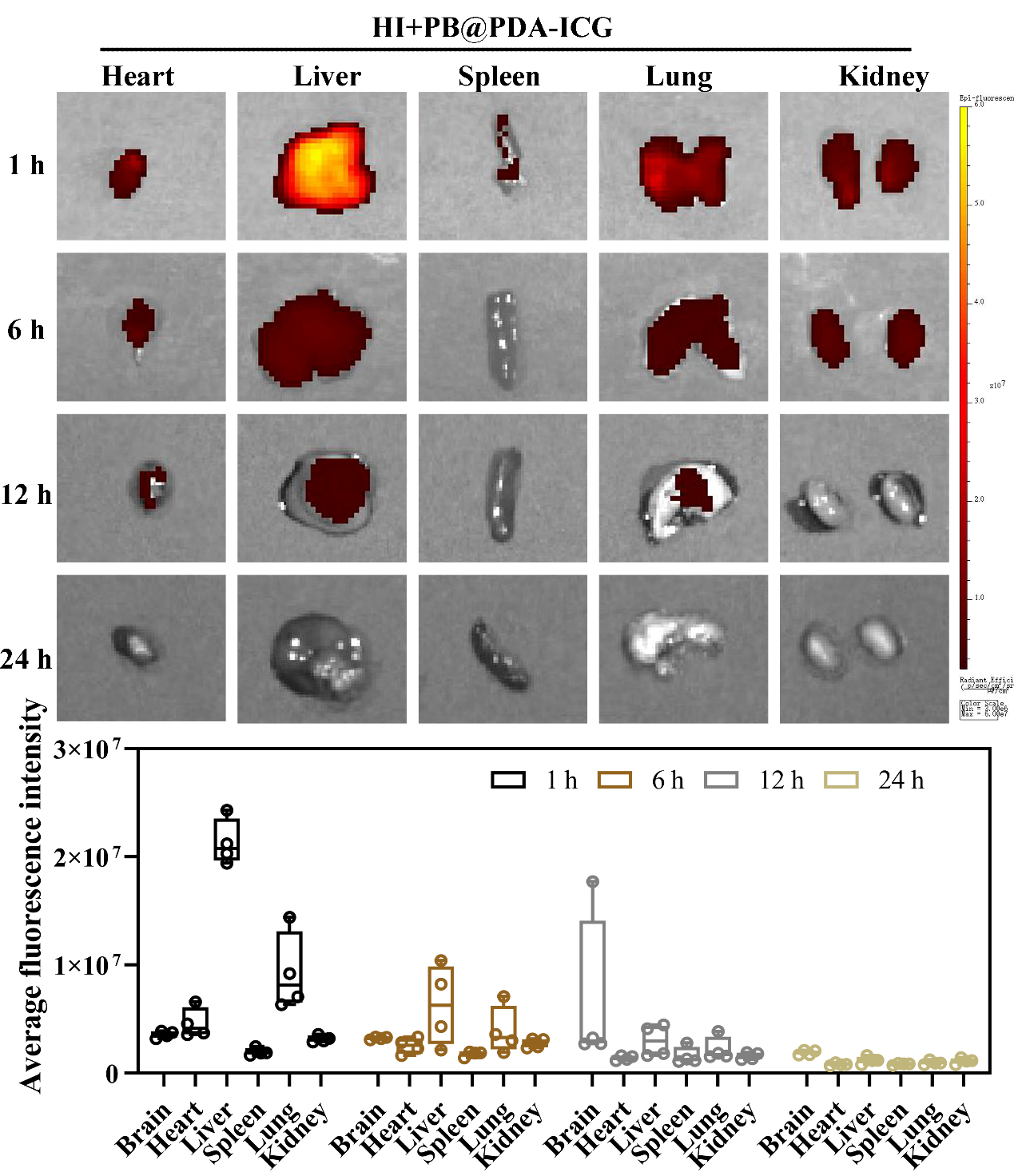
**

**Figure S10.** (A) Fluorescence imaging of brain and main organs from mice in the HI+PB@PDA group at 1 h, 6 h, 12 h, and 24 h after intracardiac injection with ICG-labeled PB@PDA NPs. (B) The fluorescence level of the main organs was quantified by the mean fluorescence intensity (Data are presented as means ± SD of 4 per group).

**
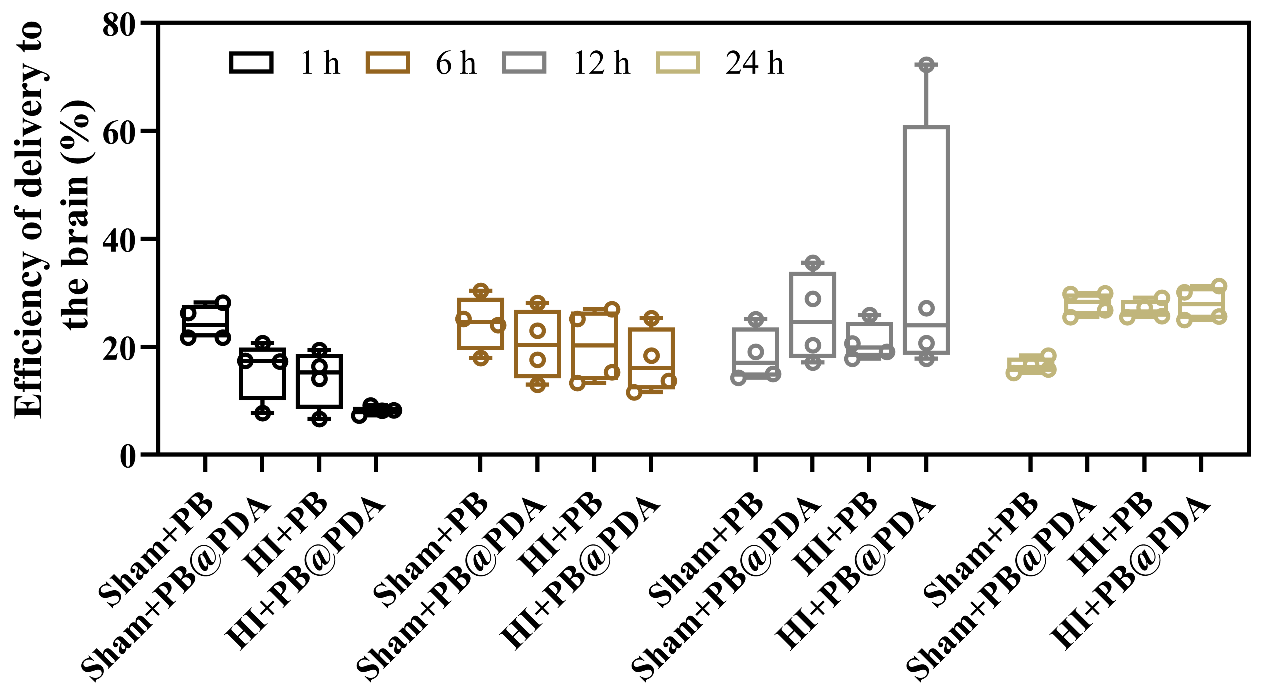
**

**Figure S11.** Efficiency of delivery to the brain from mice in the Sham+PB, Sham+PB@PDA, HI+PB, HI+PB@PDA groups at 1 h, 6 h, 12 h, and 24 h after intracardiac injection with ICG-labeled PB@PDA NPs (Data are presented as means ± SD of 4 per group).

**
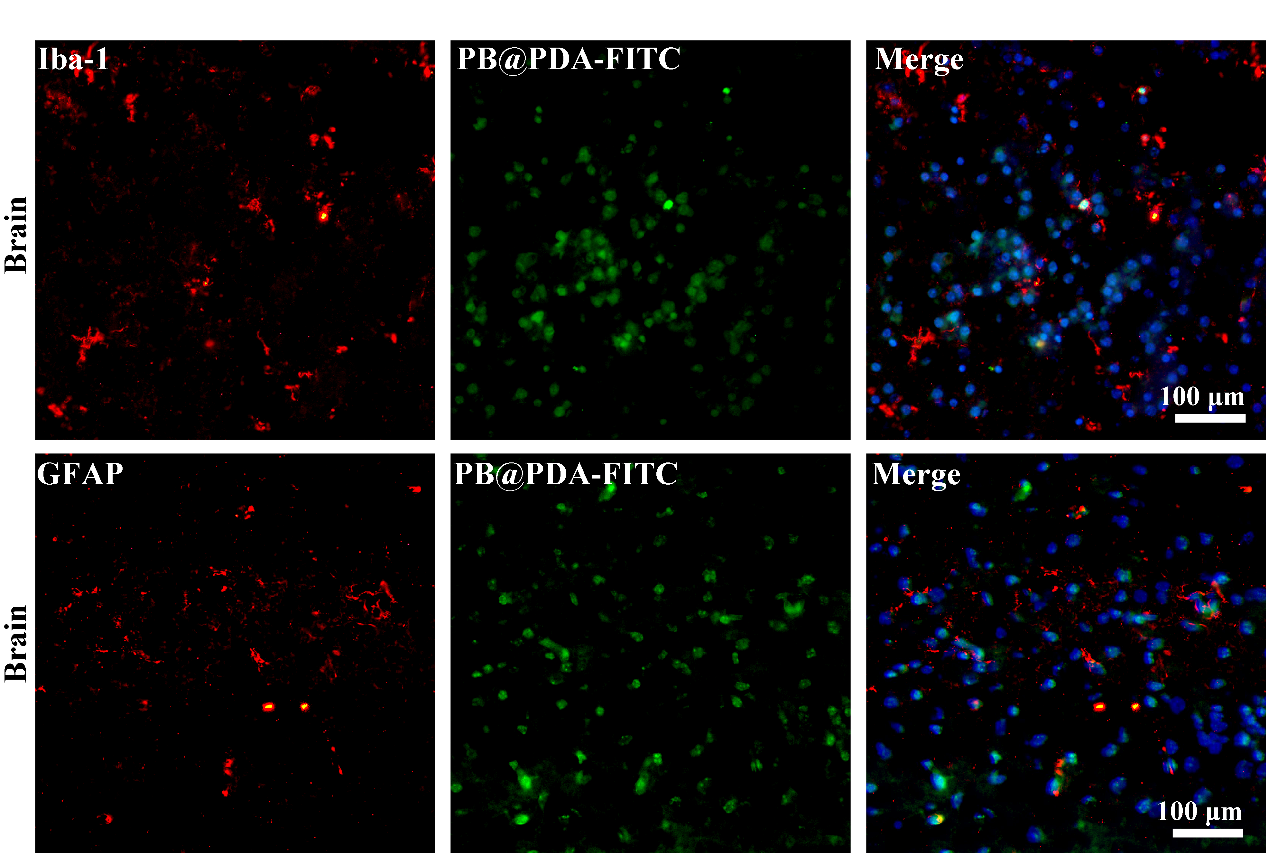
**

**Figure S12.** Representative fluorescence staining of Iba-1 and GFAP of brain section 1 h after intracardiac injection of FITC-labeled PB@PDA NPs at 1 h post-HI. DAPI (Blue, nuclei), FITC (Green, PB@PDA), Iba-1 (Red, microglia) and GFAP (Red, astrocytes). Scale bar: 100 μm.


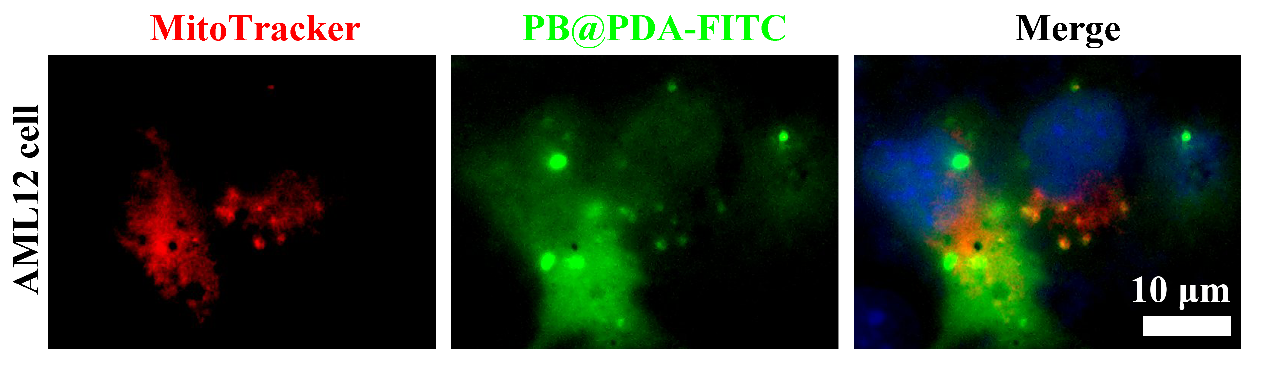


**Figure S13.** Representative images on MitoTracker Red CMXRo in AML12 cell after treatment FITC-labeled PB@PDA NPs. DAPI (Blue, nuclei), FITC (Green, PB@PDA NPs), Mito-tracker (Red, mitochondria). Scale bar: 10 μm.

**
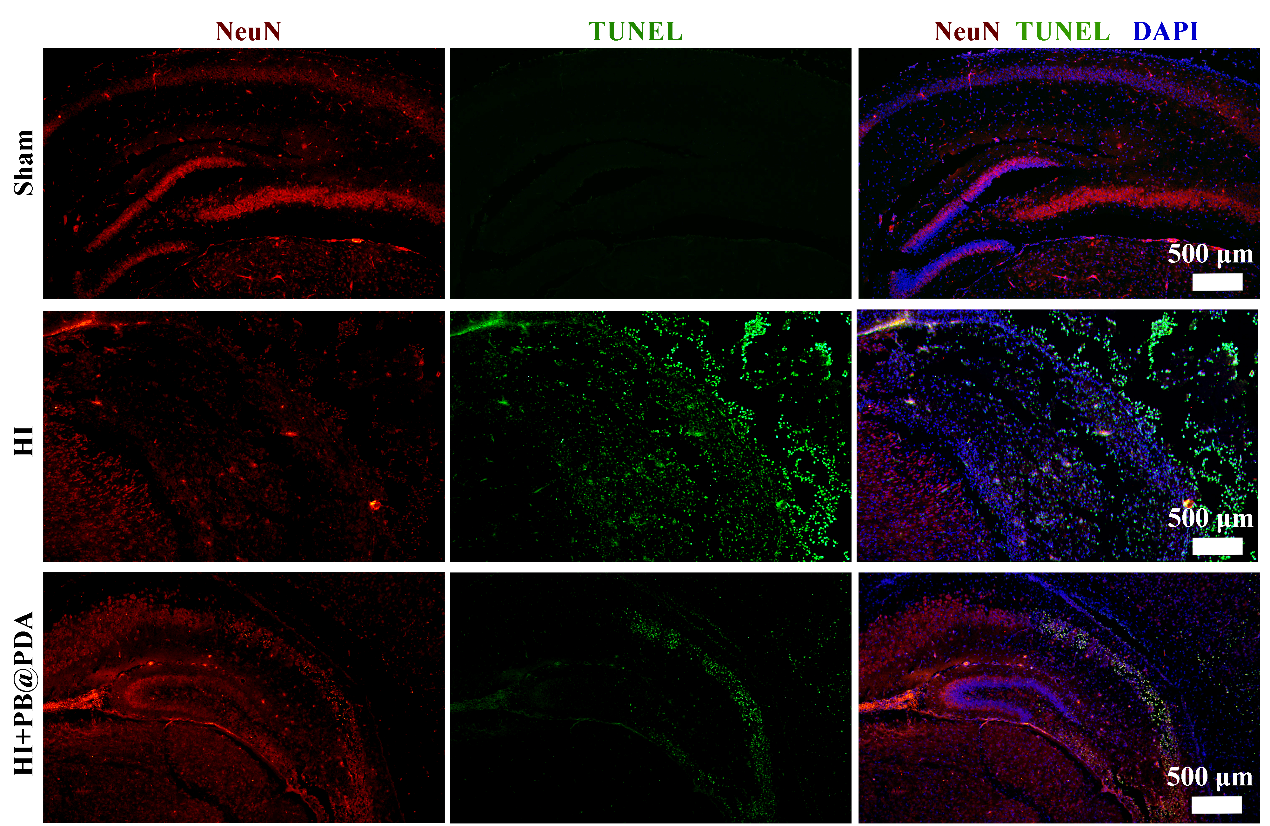
**

**Figure S14.** Representative fluorescence image of NeuN and TUNEL co-staining in hippocampus 48 h post-HI. DAPI (Blue, nuclei), TUNEL (Green, apoptosis cell), Neun (Red, neuron). Scale bar: 500 μm.

**
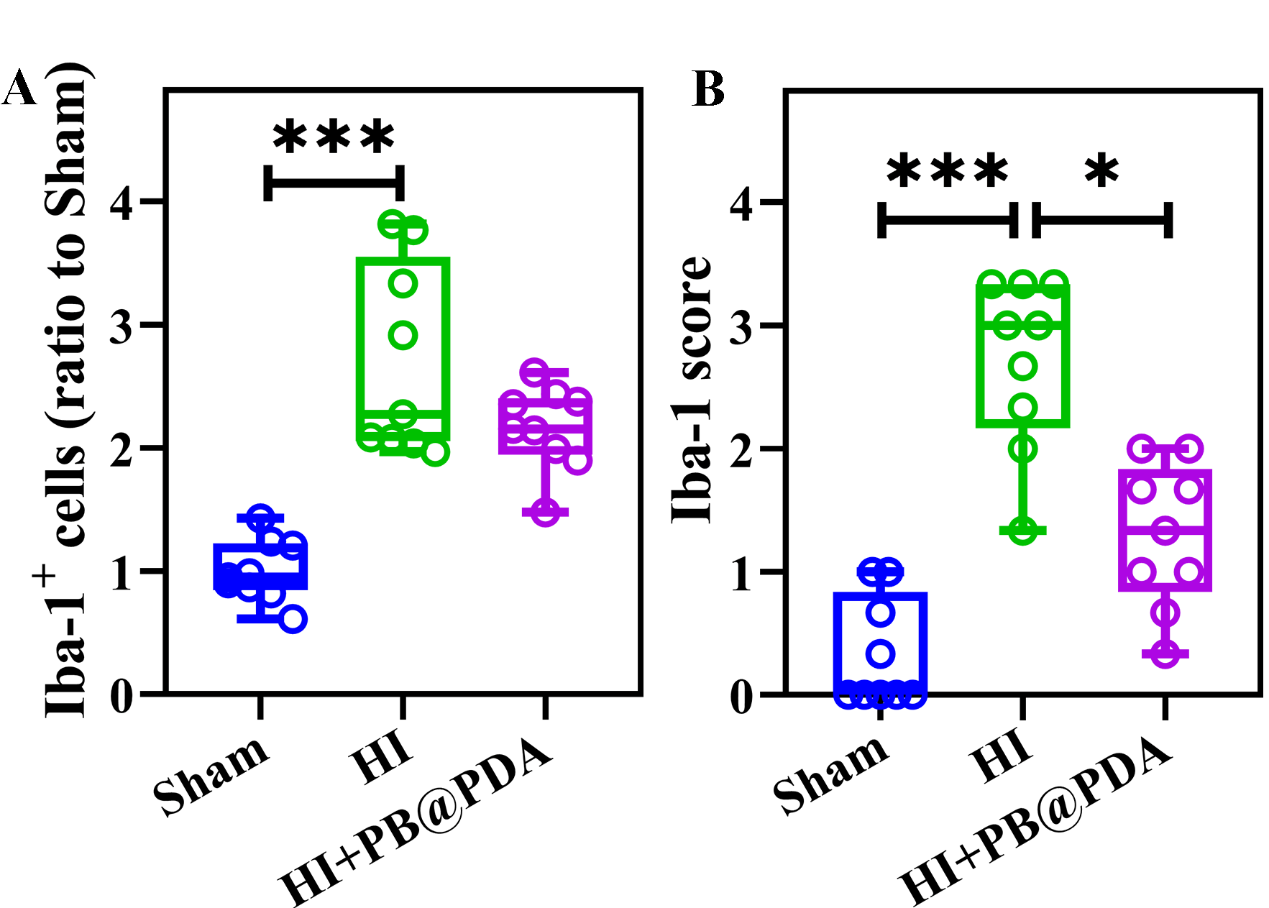
**

**Figure S15.** (A) Quantification of the number of Iba-1^+^ cell in Figure 6A (Data are presented as means ± SD of 9 per group, Kruskal-Wallis ANOVA test, ****p* < 0.001). (B) Quantification of the score of microglia in Figure 5A (Data are presented as means ± SD of 9 per group, Kruskal-Wallis ANOVA test, **p* < 0.05, ****p* < 0.001).

**
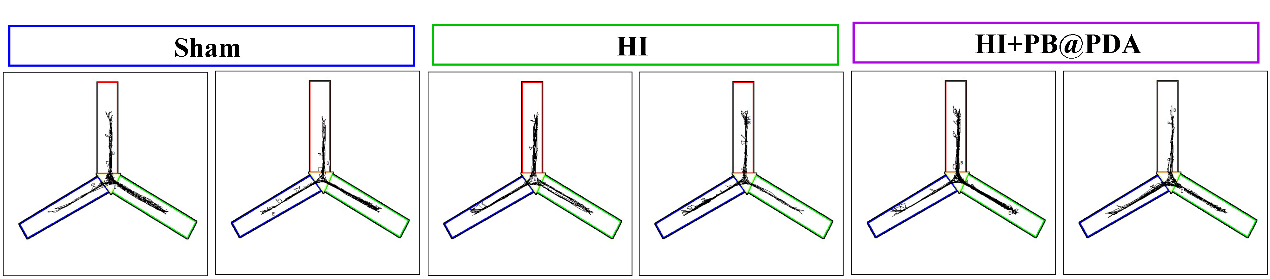
**

**Figure S16.** The path of mice in Y-maze was present as performed 28 days post HI.

**
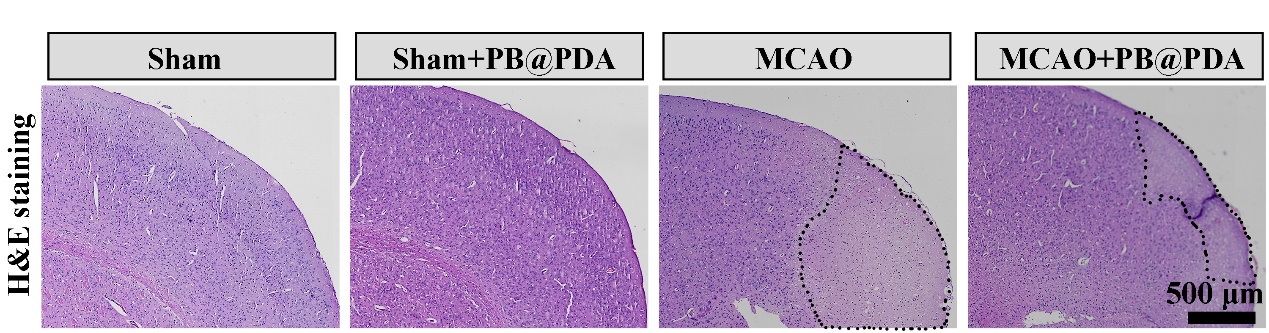
**

**Figure S17.** H&E staining of brain tissues at 24 h post-MCAO. Scale bar: 500 μm.

**Table S1. Hydrodynamic sizes and Zeta potentials of PB, PDA, and PB@PDA NPs**

| Sample | Hydrodynamic size  (nm) | Polydispersity index  (PDI) | Zeta potential  (mV) |
| --- | --- | --- | --- |
| PB | 291.2 ± 13.2 | 0.28 ± 0.04 | -8.69 ± 0.71 |
| PDA | 680.1 ± 8.9 | 0.47 ± 0.02 | -39.07 ± 1.86 |
| PB@PDA | 485.1 ± 10.8 | 0.20 ± 0.01 | -34.90 ± 1.85 |

Data are provided as mean ± SD (n = 3).

**References**

[1] RICE J E, 3RD, VANNUCCI R C, BRIERLEY J B. The influence of immaturity on hypoxic-ischemic brain damage in the rat [J]. Ann Neurol, 1981, 9(2): 131-41.

[2] XIN D, LI T, CHU X, et al. MSCs-extracellular vesicles attenuated neuroinflammation, synapse damage and microglial phagocytosis after hypoxia-ischemia injury by preventing osteopontin expression [J]. Pharmacol Res, 2021, 164: 105322.

[3] LIANG H, MATEI N, MCBRIDE D W, et al. TGR5 activation attenuates neuroinflammation via Pellino3 inhibition of caspase-8/NLRP3 after middle cerebral artery occlusion in rats [J]. J Neuroinflammation, 2021, 18(1): 40.

[4] LUBICS A, REGLODI D, TAMAS A, et al. Neurological reflexes and early motor behavior in rats subjected to neonatal hypoxic-ischemic injury [J]. Behav Brain Res, 2005, 157(1): 157-65.

[5] HU Q, MANAENKO A, BIAN H, et al. Hyperbaric Oxygen Reduces Infarction Volume and Hemorrhagic Transformation Through ATP/NAD(+)/Sirt1 Pathway in Hyperglycemic Middle Cerebral Artery Occlusion Rats [J]. Stroke, 2017, 48(6): 1655-64.
