# Supplementary figures and images for: Polydopamine-Cloaked Nanoarchitectonics of Prussian Blue Nanoparticles Promote Functional Recovery in Neonatal and Adult Ischemic Stroke Models
Source: Biomater Res. 2024 Sep 18;28:0079. doi: 10.34133/bmr.0079 (PMC11409202; doi:10.34133/bmr.0079)

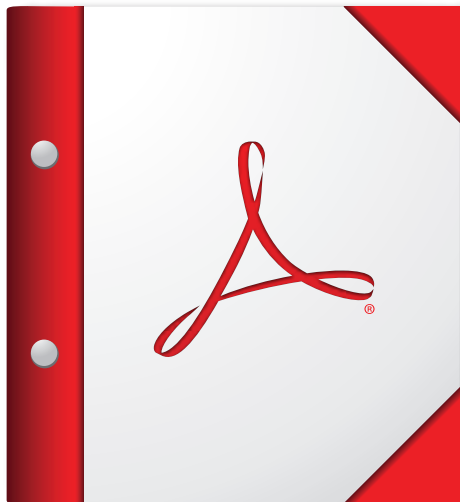

为获得最佳体验，请在 **Acrobat X**、**Adobe Reader X**  
或更高版本中打开此 **PDF** 包。

立即下载 Adobe Reader !

Supplement: Supplementary 1 — Supplementary Text Figs. S1 to S17 Table S1 References [file bmr.0079.f1.zip › BMR_LicenseToPublish2024.pdf]
